# Characterization of viruses in a tapeworm: phylogenetic position, vertical transmission, and transmission to the parasitized host

**DOI:** 10.1038/s41396-020-0642-2

**Published:** 2020-04-14

**Authors:** Megan A. Hahn, Karyna Rosario, Pierrick Lucas, Nolwenn M. Dheilly

**Affiliations:** 1 School of Marine and Atmospheric Sciences, Stony Brook University, Stony Brook, NY USA; 2 College of Marine Science, University of South Florida, Saint Petersburg, FL USA; 3 Unité Génétique Virale de Biosécurité, ANSES, Agence Nationale de Sécurité Sanitaire de l’Alimentation, de l’Environnement et du Travail—Laboratoire de Ploufragan-Plouzané, Ploufragan, France

**Keywords:** Biodiversity, Metagenomics

## Abstract

Parasitic flatworms (Neodermata) infect all vertebrates and represent a significant health and economic burden worldwide due to the debilitating diseases they cause. This study sheds light for the first time into the virome of a tapeworm by describing six novel RNA virus candidate species associated with *Schistocephalus solidus*, including three negative-strand RNA viruses (order *Jingchuvirales*, *Mononegavirales*, and *Bunyavirales*) and three double-stranded RNA viruses. Using in vitro culture of *S. solidus*, controlled experimental infections and field sampling, we demonstrate that five of these viruses are vertically transmitted, and persist throughout the *S. solidus* complex life cycle. Moreover, we show that one of the viruses, named Schistocephalus solidus rhabdovirus (SsRV1), is excreted by the parasite and transmitted to parasitized hosts indicating that it may impact *S. solidus*–host interactions. In addition, SsRV1 has a basal phylogenetic position relative to vertebrate rhabdoviruses suggesting that parasitic flatworms could have contributed to virus emergence. Viruses similar to four of the *S. solidus* viruses identified here were found in geographically distant *S. solidus* populations through data mining. Further studies are necessary to determine if flatworm viruses can replicate in parasitized hosts, how they contribute to parasite infection dynamics and if these viruses could be targeted for treatment of parasitic disease.

## Introduction

Parasitic flatworms (Phylum Platyhelminthes) have long attracted attention for their high prevalence in humans and economically important animals, including livestock and farmed fish, and for causing debilitating diseases. Trematodes, commonly known as flukes, and cestodes, known as tapeworms, are of particular interest because around 25–30% of humans are currently infected with at least one of these flatworms [1–4]. Several of the pathologies associated with fluke and tapeworm infections are considered major neglected diseases affecting countries in the Americas, Asia, and Africa. Parasitic infections can remain asymptomatic for long periods, and symptoms are often misdiagnosed, making flatworm-associated diseases difficult to target and treat [5–9]. Very few pharmaceutical products are currently available for treatment and instances of parasite resistance and allergic reactions to these drugs have been reported [10]. Thus, researchers have long sought to understand the underlying molecular mechanisms driving host susceptibility and parasite pathogenicity to develop alternative therapeutic strategies.

The parasitology field is beginning to recognize that parasite-associated microbes, including viruses, can affect parasite fitness and influence the outcome of parasitic infection, and these microbes may be targeted for treatment of parasitic diseases [11–15]. We need to characterize the virome of parasitic organisms to understand the role of parasites in virus evolution and host–microbe interactions, to determine the role of viruses in parasite virulence, and to identify patterns and processes of host–parasite–virus coevolution [14]. Viruses of parasitic flatworms remain largely unknown. The first microscopic observation of virus-like particles in a parasitic flatworm was reported by Jean-Lou Justine [16]. More recently, Shi et al. [17] studied the virome of a broad range of invertebrates and reported the complete genomes of a virus of the order *Bunyavirales* from the fluke *Schistosoma japonicum* and of a virus of the family *Nyamiviridae* in the order *Mononegavirales* from a mix of tapeworm *Taenia spp*.

**Fig. 1:**
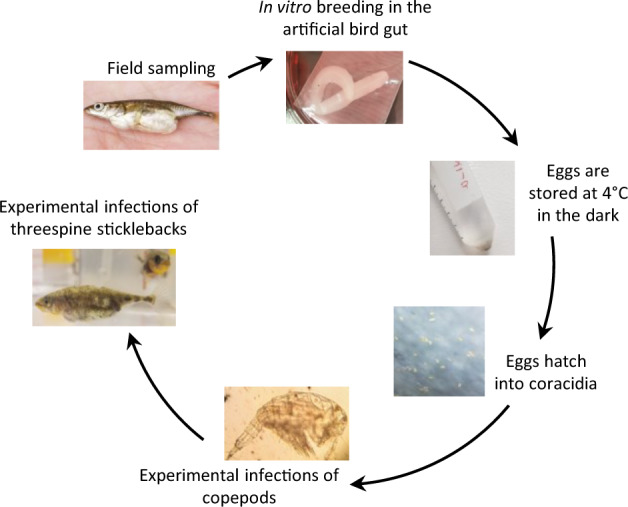
An ideal experimentally tractable system. The life cycle of *S. solidus* is reproduced in laboratory conditions to test virus transmission.

We have previously identified the tapeworm *Schistocephalus solidus* as an ideal experimentally tractable system to investigate host–parasite–microbe interactions [11]. *S. solidus* has a complex life cycle in which definitive hosts are fish-eating birds and intermediate hosts are a range of cyclopoid copepods and threespine sticklebacks (*Gasterosteus aculeatus*) [18, 19]. In vitro culture of *S. solidus* facilitates experimentation (Fig. 1) [20]: eggs are conserved in the fridge and hatch into coracidia that are used to infect copepods, that are themselves used to infect sticklebacks [21]. *S. solidus* has a broad geographic distribution throughout the Northern Hemisphere that parallels the distribution of its stickleback host, providing researchers with an exceptional playground to answer questions that relate to the ecology and evolution of host–parasite interaction [22–24].

Herein, we investigate the presence of viruses in *S. solidus* from Alaska and report the discovery of three negative-strand and three double-stranded RNA viruses. We also test the prevalence and vertical transmission of identified viruses and evaluate the possibility of cross-species transmission to parasitized hosts through in vitro culturing, experimental infections, and field sampling.

## Materials and methods

A detailed “Material and methods” section is available as Supplementary information and summarized in Fig. S1.

### Initial virus discovery

Viruses were purified from four *S. solidus* plerocercoids field-collected in June 2016 from Cheney Lake, Alaska (61° 12′ 17″ N, 149° 45′ 33″). RNA was sequenced, and viral genome fragments were assembled and identified as described previously [25].

### Sampling and in vitro culture of *S. solidus*

In June of 2018, 31, 20, and 46 plerocercoids were collected from sticklebacks fished in Cheney Lake, Wolf Lake (61° 38′ 36″ N, 149° 16′ 32″ W), and Loberg Lake (61° 33′ 33.5″ N 149° 15′ 28.9″ W), respectively, and preserved in RNA later. Both plerocercoids and fish liver were collected for 24 sticklebacks.

In addition, freshly collected mature plerocercoids were bred in vitro by incubating size-matched pairs in culture medium [20, 21, 26]. Eggs collected after 48 h and 96 h were pooled, washed in sterile water and conserved at 4 °C in darkness for up to a year. Parent parasites and a sample of culture medium were conserved in RNAlater. We obtained eggs from 16 families from Cheney lake, 13 families from Wolf lake, and 9 families from Loberg lake, where a family refers to eggs collected from the breeding of one parasite pair. To stimulate egg hatch, a small aliquot of the stock of eggs were warmed and exposed to light [20]. Newly hatched coracidia were either used for experimental infections, processed for virus purification, or transferred to RNAlater.

### Experimental infections

C5 copepodite stages of a highly susceptible strain of *Macrocyclops albidus* from Norway were exposed to one coracidium each [27]. Fourteen days postexposure, copepods were screened under the microscope to assess infection success, and were individually sorted, rinsed in sterile water and transferred to RNAlater. To ensure the absence of *S. solidus* in exposed but nonconspicuously infected copepods, we conducted PCRs with *S. solidus* specific primers [28].

In June 2018, we also collected mature males and gravid females of threespine sticklebacks, from Rabbit Slough (61° 32′ 08.1″ N 149° 15′ 10.0″ W), Cheney lake, and Loberg lake and completed crosses in vitro. Fish were reared in the laboratory at 18 °C and 16:8 light:dark cycle for 5-months before being singly exposed to copepods parasitized with a single virus(+) *S. solidus*. Fish were maintained in large tanks for 8 weeks, before plerocercoids, fish body cavity, spleen, head kidney, liver, and intestine were collected and preserved in RNAlater.

### Virus purification from coracidia

Coracidia from *S. solidus* families from Wolf Lake, Loberg Lake, and Cheney Lake were used for virus purification before conducting a second sequencing effort (Table S1). PCR assays were used to select families infected by viruses (see below). Coracidia were homogenized in sterile suspension medium buffer through bead beating in a Fisherbrand Bead Mill 4 Homogenizer for 1 min using a mixture of glass beads. Virus particles were purified from homogenates through 0.45 μm filtration and nuclease treatment following methods used to characterize virions isolated from arthropods [29].

### RNA extractions

RNA from purified viruses and total RNA from culture medium, coracidia, copepods, and stickleback tissues were extracted using the RNeasy kit with the on-column DNase digestion step following manufacturer’s recommendations. Total RNA from plerocercoids was extracted using Trizol^TM^ reagent following the manufacturer’s recommendations and DNase treated with Turbo DNA free kit.

### Sequencing and virus discovery (second round)

Total RNA extracts from plerocercoids and coracidia, and RNA extracts from the purified viral fraction were processed for virus discovery. cDNA was obtained using the SuperScript IV First Strand Synthesis System with random hexamers followed by second-strand cDNA synthesis using the Klenow Fragment DNA polymerase and cleaned using the ZR DNA Clean & Concentrator kit. Purified cDNA samples from the viral fraction (V) and from total RNA (T) were pooled into two samples (Table S1) and fragmented to 300 bp using a Covaris M220 instrument at the Molecular Genomics Core at the H. Lee Moffitt Cancer Center & Research Institute. Library construction was performed with the Accel-NGS 1S Plus DNA Library Kit for Illumina Platforms following manufacturer’s instructions using 18 cycles of dual indexing PCR for the V-library, which had low cDNA input (<1 ng/μl), and 10 cycles for the T-library (cDNA input > 10 ng/ul). Both libraries were commercially paired-end sequenced (2 × 150 bp) on an Illumina HiSeq 4000 System at GENEWIZ.

Raw sequences were processed using Trimmomatic version 0.36.0 [30] with default parameters, except for a read head crop of 10 bp. Sequence quality was then verified with FastQC version 0.11.5 [31]. Quality-filtered sequences from the V-library were assembled following a pipeline for PCR amplified libraries [32]: sequences were dereplicated using the Clumpify tool from the BBtools package (sourceforge.net/projects/bbmap/) and assembled using single-cell SPAdes [33]. Quality-filtered sequences from the T-library were assembled with RNAspades. Viral contigs were identified using BLASTx (*e* value < 10^−10^) against the NCBI Reference Sequence database (RefSeq Release number 93, https://www.ncbi.nlm.nih.gov/refseq/).

### Viral genome completion

Quality-filtered reads and contig sequences associated with each of the viruses were retrieved by comparing sequences through BLASTn to a database containing all newly identified contig sequences and closely related sequences. All reads and contigs were reassembled using the default overlap-consensus algorithm implemented in Geneious version R7 resulting in near-complete genome sequences.

RNA extracts from virus(+) coracidia were used for PCR and rapid amplification of complimentary ends (RACE) assays to close genome gaps, sequence genomes ends, and confirm genome topology (primers listed in Tables S2 and S3) [34, 35]. All RACE products were cloned using the CloneJET PCR Cloning Kit and sequenced using vector primers. All PCR and cloned RACE products were Sanger sequenced by TACGen.

### Virus genome characterization and phylogenetic analysis

Open reading frame (ORF) prediction was performed using Translate on ExPASy (https://www.expasy.org/). Annotation of domains was deduced from comparisons against the Conserved Domain Database as implemented by BLASTp against the nr protein database. Initial supergroup assignments were determined from best BLAST matches. Predicted RNA dependent RNA polymerase (RdRP) sequences from *S. solidus*-associated viruses, and representative sequences from related viral families and genera ratified by the ICTV and from recent metatranscriptomic studies [36–39], were aligned using the E-INS-I algorithm implemented in the program MAFFT (version 7) [40]. Next, all ambiguously aligned regions were removed using TrimAl (version 1.2) [41]. For each dataset, the best-fit model of aa substitution was determined using Smart Model Selection [42]. Phylogenetic trees were inferred using the maximum likelihood method implemented in PhyML (version 3.0) [43] using the best-fit model and best of NNI and Subtree Pruning and Regrafting branch swapping. Support for nodes on the trees were assessed using an approximate likelihood ratio test with the Shimodaira–Hasegawa-like procedure.

### PCR assays to assess *S. solidus* virus presence

The first strand cDNA was synthesized by reverse transcribing 500 ng of total RNA with random hexamers and RevertAid H Minus Reverse Transcriptase, as per manufacturer’s recommendations. Polymerase chain reaction was conducted using the Advantage 2 PCR system using primers targeting the RdRP gene of each virus (Table S2). Amplicon presence was assayed on 1% agarose gel with SyBR Safe. Select PCR products were Sanger sequenced to confirm primer specificity.

### Data mining

To assess virus presence in other populations, we queried publicly available transcriptomes. BLASTn searches were used to identify reads that align to the newly identified viruses in data from PRJEB7355 (2 biosamples of wild-caught Norwegian and German *S. solidus*) and PRJNA304161 (15 biosamples from Clatworthy reservoir, England, UK [44]). Sequence data from PRJNA304161 were downloaded and processed as follow: reads were trimmed with the Trimmomatic version 0.36 [30] with default settings. Quality-filtered reads were aligned against the *S. solidus* reference genome (GCA_900618435.1) with Bowtie2 (version 2.3.4.1) [45]. Unmapped reads were collected using SAMtools 1.8 [46] and bedtools [47] and assembled using the shovill method (https://github.com/tseemann/shovill). Viral contigs were identified using BLASTx as described above.

## Results

### Discovery of novel candidate virus species

Two high-throughput sequencing efforts, combined with PCR and RACE, allowed us to assemble the genomes of six unique viral candidate species associated with *S. solidus*. BLAST searches against the genome of *S. solidus* did not yield significant matches, confirming that the viruses are not endogenous.

**Fig. 2:**
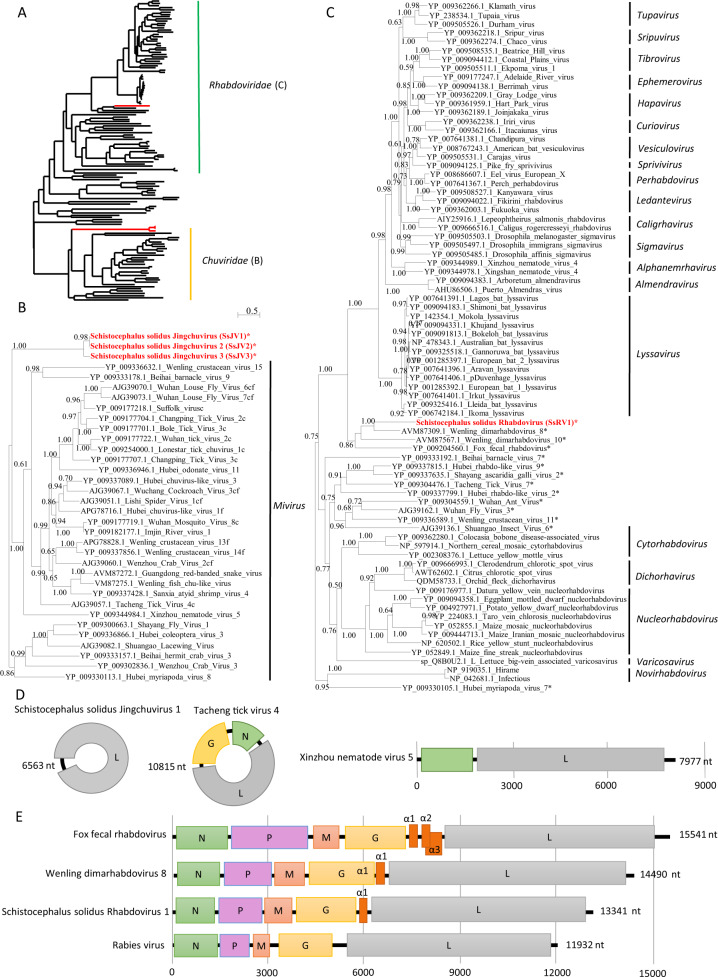
Phylogenetic and genomic characterization of SsRV1 and SsJV1. **a** Phylogenetic analysis of the RdRP of viruses from the order *Mononegavirales* and *Jingchuvirales*. The tree was inferred with PhyML using the LG substitution model. **b**, **c** close up views of the phylogenetic tree of the RdRP of viruses from the families *Chuviridae* and *Rhabdoviridae*, respectively. Values next to the branch indicate the results of a Shimodaira–Hasgawa branch test. Genus and family names are provided next to the branches. Asterisk (*) indicates unassigned viruses. c indicates circular genomes. f indicates fragmented genomes. **d**, **e** Genome organization of viruses from *S. solidus* compared with the genome of closely related viruses of the families *Chuviridae* and *Rhabdoviridae*, respectively. Boxes represent putative genes. The black line indicates noncoding regions.

The first virus, named Schistocephalus solidus Rhabdovirus 1 (SsRV1; accession number MN803433), showed a maximum of 59% amino acid (aa) identity to the RdRP of unassigned and partially sequenced Bat Rhabdovirus (AIF4284.1) from the family *Rhabdoviridae*, order *Mononegavirales*. The SsRV1 genome encodes the five canonical proteins N-P-M-G-L with an additional short protein between G and L (Fig. 2e). All identified ORFs were flanked by conserved transcription initiation (UUGU) and transcription termination/polyadenylation sequences (UC[U]^7^) with very short intergenic region (Table S4). The L protein included the *Mononegavirales*-like RdRP domain (pfam 00946), the *Mononegavirales* mRNA capping region V (pfam 14318), a paramyxovirus-like mRNA capping enzyme (TIGR04198), and a *Mononegavirales* virus-capping methyltransferase (pfam 14314).

The second virus, named Schistocephalus solidus Jingchuvirus 1 (SsJV1; accession number MN803434), had a circular genome encoding a single protein (Fig. 2d) with a maximum of 28% aa identity to the L protein of Hubei Myriapoda virus 8 (YP_009330113.1) of the order *Jingchuvirales*. The predicted protein possesses a *Mononegavirales* RdRP domain (pfam 00946), a paramyxovirus mRNA capping enzyme (TIGR04198) and the *Mononegavirales* virus-capping methyltransferase (pfam 14314).

We inferred a phylogenetic tree using the predicted RdRP sequences from SsRV1, SsJV1, and representative members of the orders *Jingchuvirales* and *Mononegavirales* (Figs. 2a and S2). Sequences clustered following established genera and families ratified by the ICTV, except for *S. solidus*-associated viruses, which grouped into distinct clades (Figs. 2a and S1). Our results show that SsJV1 belongs to the order *Jingchuvirales*, and likely represents a new taxon within the family *Chuviridae* (Fig. 2b). SsRV1 belongs to the family *Rhabdoviridae*, grouping closely with viruses reported from metatranscriptomic studies and whose host association remains unknown, including bat rhabdovirus, fox fecal rhabdovirus, Wenling dimarhabdovirus 8, and Wenling dimarhabdovirus 10 [48, 49]. Notably, SsRV1 and these closely related viruses represent a new taxon basal to *Lyssavirus* and to the dimarhabdovirus supergroup (Fig. 2c), with genomes of variable length, and characterized by the presence of one to three small proteins in the region between G and L (Fig. 2e).

**Fig. 3:**
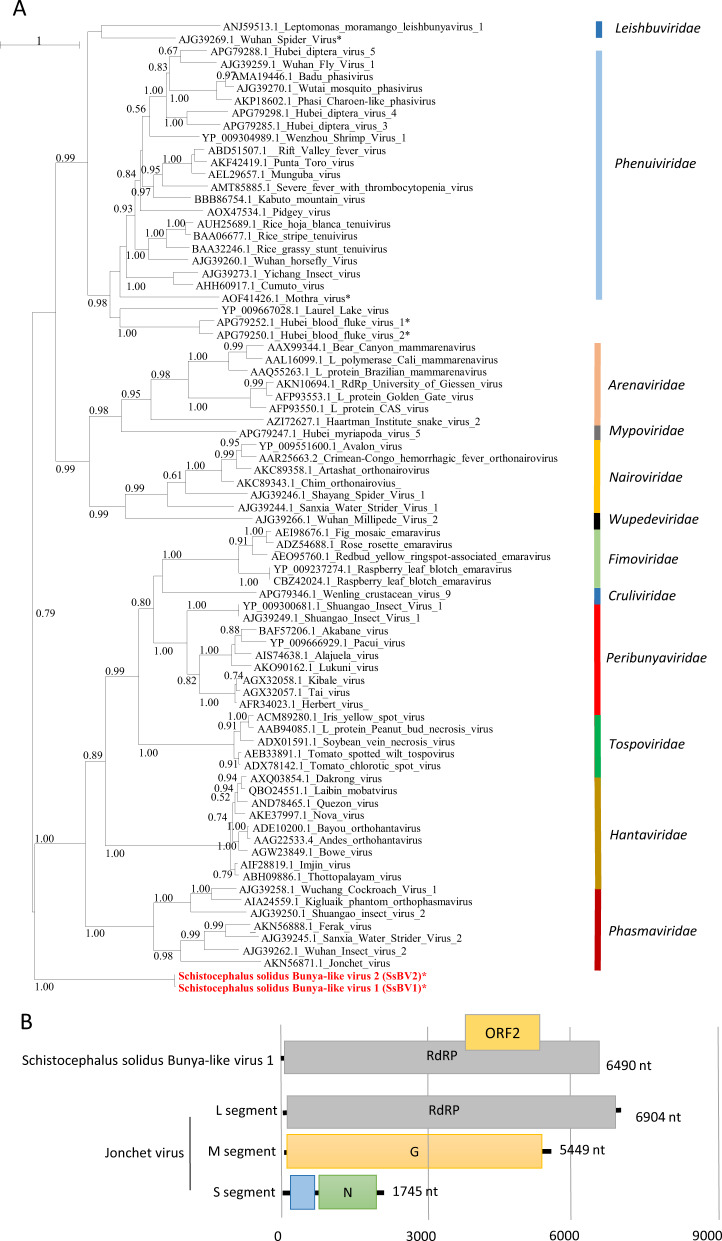
Phylogenetic and genomic characterization of SsBV1. **a** Phylogenetic analysis of the RdRP of viruses of the order *Bunyavirales*. The tree was inferred with PhyML using the LG substitution model. Values next to the branch indicate the results of a Shimodaira–Hasgawa branch test. Asterisk (*) indicates unassigned viruses. Family names are provided next to the branches. **b** Genome organization of *S. solidus* bunya-like virus aligned to the genome of a related virus.

The third viral genome, named Schistocephalus solidus bunya-like virus 1 (SsBV1; accession number MN803432), had a maximum of 36% identity to the RdRP of the Beihai barnacle virus 5 (APG79235.1). The longest predicted ORF (Fig. 3b) possesses a bunyavirus RdRP domain (pfam04196). Notably, SsBV1 was only found in sequencing data from the total RNA library and PCR assays confirmed its absence in samples that went through viral purification. We inferred a phylogenetic tree using SsBV1 and the L segment of representative members of all assigned families within the order *Bunyavirales*. The tree confirmed that SsBV1 has no known relatives and likely constitutes a new family of viruses (Fig. 3a).

**Fig. 4:**
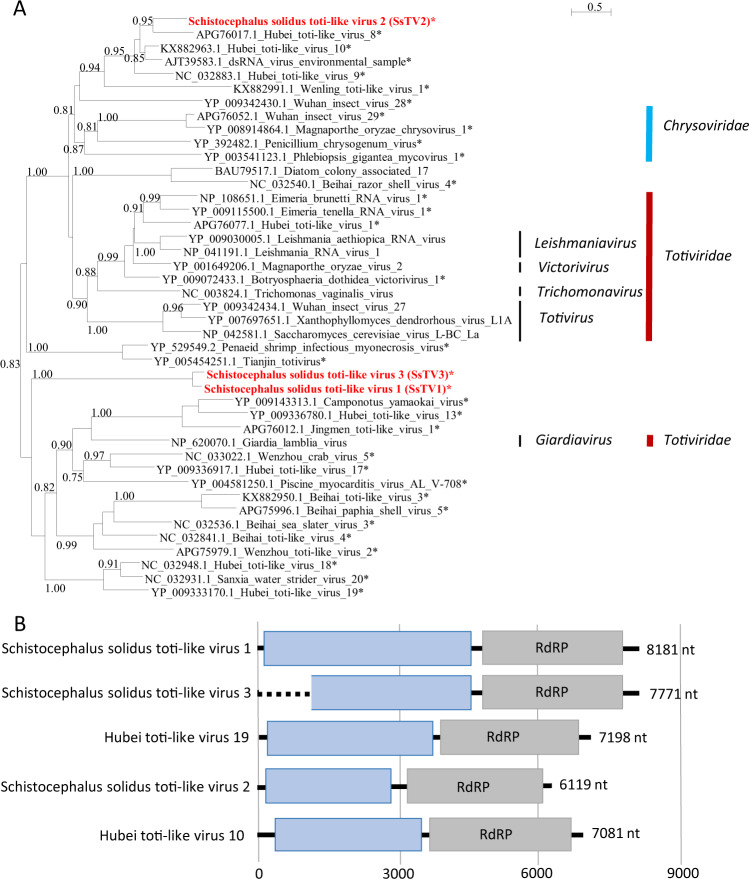
Phylogenetic and genomic characterization of SsTV1, SsTV2, and SsTV3. **a** Phylogenetic analysis of the RdRP of dsRNA viruses. The tree was inferred with PhyML using the LG substitution model. Values next to the branch indicate the results of a Shimodaira–Hasgawa branch test. Asterisk (*) indicates unassigned viruses. Genus and family names are provided next to the branches. **b** Genome organization of toti-like viruses from *S. solidus* aligned to the genome of closely related viruses.

Finally, three sequences similar to dsRNA viruses of the families *Totiviridae* and *Chrysoviridae* (Fig. 4) were found and named Schistocephalus solidus toti-like viruses (SsTV1, SsTV2, and SsTV3; accession numbers MN803435 through MN803437). These viral sequences showed the highest similarities to the partial RdRP sequence of Dumyat virus (QAY29251.1, SsTV1, 27% aa identity and SsTV3, 28% aa identity), or to Hubei toti-like virus 10 (YP_009336493.1, SsTV2, 36% aa identity). All three viruses had two ORFs (Fig. 4b), with the second protein encoding for an RdRP similar to Luteovirus, Totivirus, and Rotavirus (pfam02123). We inferred a third phylogenetic tree using SsTV1, SsTV2, and SsTV3 together with 40 viruses representing the families *Totiviridae*, *Chrysoviridae*, and unassigned members closely related to these families (Fig. 4). Phylogenetic analyses revealed that SsTV1 and SsTV3 cluster together and are most closely related to viruses discovered in other invertebrates including Lophotrochozoa, Nematoda, Crustacea, and Insecta, whereas SsTV2 was most closely related to viruses discovered in insects.

### Virus detection in geographically distant *S. solidus* populations through data mining

At the time of this study, two transcriptomic studies of *S. solidus* were publicly available. BLASTx searches against sequences from individuals from Germany and Norway ([50], PRJEB7355, two biosamples) revealed the presence of related strains of SsJV1 and SsTV1 in one dataset (ERX589070) and of SsTV2 in two datasets (ERX589070 and ERX589072). BLASTx searches against the de novo assembled transcriptome of *S. solidus* from Clatworthy Reservoir in Somerset, England (TSA; PRJNA304161, 15 individuals) [44] revealed two contigs representing viruses, named here SsJV2 and SsJV3, with high similarity to SsJV1 (Fig. 2). SsJV2 (GEEE01006270.1) corresponded to the full-length sequence of a variant with 94% aa sequence identity to the RdRP of SsJV1. SsJV3 (GEE01008921.1) covered only a part of the RdRP, and shared 63% aa sequence identity with SsJV1.

We further investigated the presence of viruses within the samples from England by analyzing raw sequencing reads. Viral contigs were mostly assembled from three of the 15 individuals (A12, SRR2966898, I98-2, SRR2966894, and A07, SRR2966897; Fig. S3). In addition to the above-mentioned SsJVs, we assembled a full-length sequence of the L segment of a bunya-like virus, named here SsBV2, whose genome shares 97.5% aa identity with SsBV1 (Fig. 3). Partial sequences from toti-like viruses covering 43% and 31% of the SsTV2 genome were identified and named SsTV4 and SsTV5, respectively. The consensus sequences displayed 95 and 52% aa identity to ORF1 of SsTV2. Finally, we assembled the genomes of two additional viruses, unrelated to *S. solidus* viruses from Alaska, which show 40.7% aa identity to the RdRP of Tapwovirus (Fig. S2). The two novel viruses, named SsNV1 and SsNV2 for Schistocephalus solidus Nyami-like virus share 71% nucleotide identity. Virus abundance varied substantially with viral reads representing between 0.0001 and 0.057% of the total number of reads. The most abundant viruses, all found in adult worms, were SsBV2, SsNV1, and SsJV2 with 85, 50, and 24 transcripts per millions of reads, respectively (Fig. S3).

### Prevalence and transmission mode

**Fig. 5:**
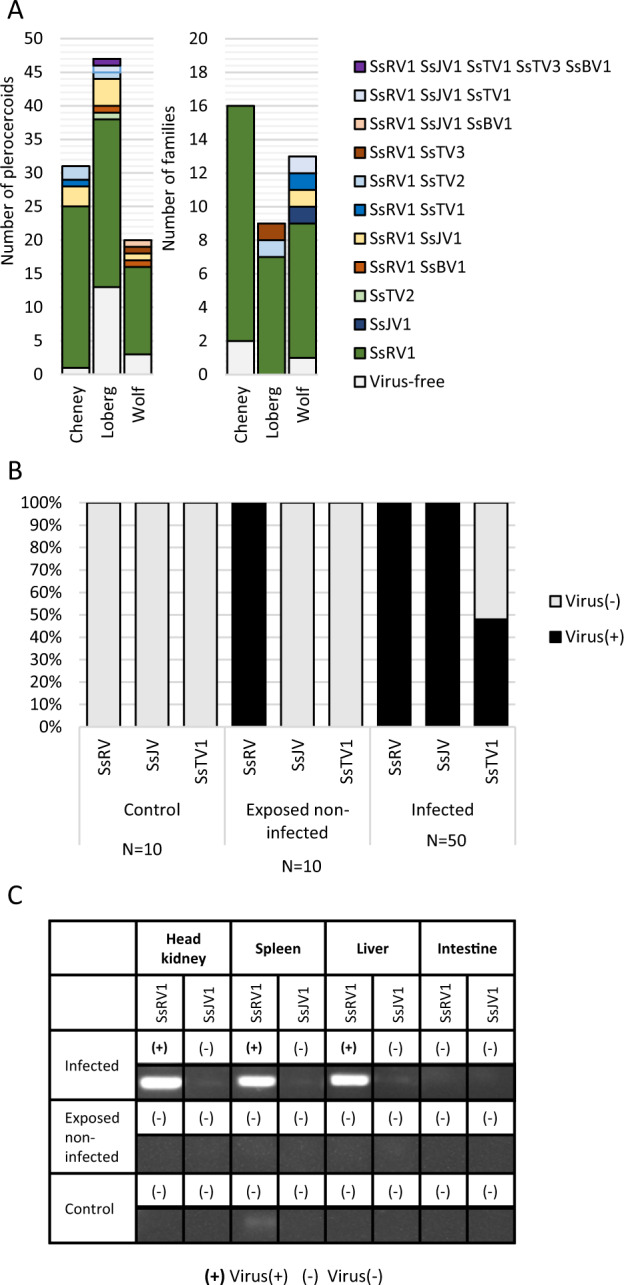
Virus prevalence and transmission over the course of *S. solidus* life cycle. **a** Virus prevalence in plerocercoids from field-collected sticklebacks and in coracidia from in vitro generated families. **b** SsRV1, SsJV1, and SsTV1 presence was assessed in copepods experimentally exposed and infected by *S. solidus*. **c** SsRV1 and SsJV1 presence was assessed in tissues of sticklebacks experimentally exposed and infected by *S. solidus*.

We tested virus prevalence in plerocercoids from field-sampled sticklebacks from three lakes in Alaska (Figs. 5a and S4–S10). SsRV1 was the most prevalent, with an overall prevalence of 81% (±7%). In contrast, SsJV1 was detected in 10% (±6%) of plerocercoids, SsTV2 and SsBV1 were each detected in 4% (±4%) of plerocercoids, and SsTV1 and SsTV3 had a prevalence of only 2% (±2%). Only 17.5% (±7%) of plerocercoids across all populations were free of all tested viruses. A Chi-square test revealed different prevalence of SsRV1 (*P* < 0.01) and of virus-free individuals (*P* < 0.05) among populations (Figs. 5a and S3). For all viruses, we observed instances where virus(+) and virus(−) plerocercoids coinfected the same stickleback host: SsRV1 (six instances), SsJV1 (two instances), SsBV1 (one instance), SsTV1 (two instances), SsTV2 (four instances), and SsTV3 (two instances) (Figs. 5a and S4–S10).

We tested virus presence in coracidia hatched from eggs collected upon in vitro breeding. Thirty-four families were SsRV1(+), four families were SsJV1(+), one family was SsTV1(+), one family was SsTV2(+), and one family was SsTV3(+), indicating that these viruses are vertically transmitted (Figs. 5a, S4 and S11). None of the plerocercoids used for breeding was SsBV1(+), preventing us from testing the virus transmission. To estimate the rate of vertical transmission, we experimentally infected copepods with individual coracidia hatched from virus(+) families. The presence of viruses in procercoids was assessed for 50 individuals (Fig. 5b). SsTV2(+) and SsTV3(+) families had very low hatching success, preventing us from running this experiment. SsRV1 and SsJV1 were found in all 50 tested procercoids, indicating a 100% success of vertical transmission of both viruses (Figs. S12 and S13). In contrast, 48% of the procercoids were infected by SsTV1 (Fig. S14).

### Cross-species transmission to parasitized hosts

To assess the potential for *S. solidus* viruses to be transmitted to *S. solidus* intermediate and definitive hosts, we tested virus presence within the secretory products of *S. solidus*. We found SsRV1 in culture medium used for breeding all 12 SsRV1(+) families, whereas SsJV1, SsTV1, SsTV2, or SsTV3 were not found in media (Fig. S19).

To assess virus transmission in the first intermediate host, we tested SsRV1, SsJV1, and SsTV1 presence in copepods exposed to virus(+) parasites, but unsuccessfully infected by *S. solidus*. The absence of *S. solidus* in these copepods was confirmed by targeted PCR (Fig. S16). SsRV1 was found in exposed but noninfected copepods indicating cross-species virus transmission from *S. solidus* to copepods (Figs. 5b and S15). No virus was found in control nonexposed copepods. We did not test virus presence in infected copepods because we could not ensure the absence of contamination from *S. solidus* upon dissection.

To assess virus transmission to the second intermediate host, we tested SsRV1 and SsJV1 presence in experimentally exposed sticklebacks (Fig. 5c). Because SsTV1 success of vertical transmission was <100%, this virus was excluded from the experiment. No virus was found in tissues of sticklebacks exposed but noninfected by *S. solidus* (four tested individuals). Four sticklebacks successfully infected by an SsRV1(+) parasite carried the virus within their liver, spleen, and head kidney, but the virus was absent from the fish gut (Fig. S17). The one fish that was successfully infected by an SsJV1(+) parasite did not transmit the virus to its fish host (Fig. S17).

To further assess virus transmission to parasitized sticklebacks, we tested virus presence in the liver of field-samples sticklebacks. SsRV1 was found in the liver of the 19 sticklebacks parasitized by SsRV1(+) parasites but it was absent from the 5 sticklebacks parasitized by SsRV1(−) parasites. Six sticklebacks were coinfected by both SsRV1(+) and SsRV1(−) parasites, and SsRV1 was found in the liver of all sticklebacks parasitized by at least one SsRV1(+) parasite (Fig. S18). None of the other viruses was found in the liver of field-sampled sticklebacks.

## Discussion

### A glimpse into the virome of a tapeworm

In the current study, we used relatively few individuals from three lakes from a restricted geographic area, the Matsu Valley in Alaska, and discovered six new candidate virus species. It is likely that sequencing viruses from a greater number of individuals, and extending the geographic area, will reveal the presence of a greater diversity of *S. solidus*-associated virus species. *S. solidus* can be found in freshwater lakes throughout the Northern Hemisphere [23, 51]. The parasite displays a great genetic diversity, mediated mostly by geographic isolation, but also by selection pressures imposed by the stickleback host [52]. It results in distinct populations in individual lakes and different parasite clades that coexist on a given continent [52]. Due to their short generation time, viruses can rapidly diverge when physically isolated in different host populations, which could lead to an enormous viral diversity with different strains of the same virus infecting parasites from distinct populations. Mining the *S. solidus* transcriptomic data generated from few individuals from England and Germany, revealed the presence of related species of jingchuviruses, bunya-like viruses, and toti-like viruses. This confirms that *S. solidus* populations on other continents are infected by different strains of the same viruses reported here. *S. solidus* most likely hosts a much greater diversity of viruses than those identified here considering that we only sampled a tiny fraction of the parasite’s geographic range and genetic diversity. This is exemplified by the detection of Nyami-like viruses from *S. solidus* populations in England.

### Divergent viruses limit sequence-based viral surveys of flatworms

While the full-length genomes of SsRV1, SsTV1, SsTV2, and SsTV3 were obtained, it is unclear whether SsJV1 and SsBV1 genomes are complete. Both assemblies revealed a single fragment that encodes the RdRP. SsJV1 belongs to order *Jingchuvirales* which includes viruses that have small, often segmented, and sometimes circular genomes [53, 54]. SsBV1 belongs to the order *Bunyavirales* that also includes viruses with segmented genomes, consisting of two to six fragments [55]. SsBV1, was only discovered in total RNA, which may indicate its sensitivity to nuclease treatment, or the absence of capsid. However, it is very likely that additional fragments exist but are highly divergent from known viruses, hindering our ability to detect them through sequence similarity-based searches. Future studies aiming at characterizing the complete genome sequences of SsBV1 and SsJV1 could use proteomics on purified virions to extract protein sequence information, which can then be used to identify viral genome sequences.

### Basal phylogenetic position of *S. solidus* viruses

Parasites have a close and intimate relationship with their hosts that could favor virus host shifts. Multi-host parasites, such as parasitic flatworms, have the potential to acquire or transmit viruses from and to each of their hosts, thus providing the means for viruses to complete major host shifts across distantly related host taxa. Phylogenetic analyses revealed that all discovered *S. solidus* viruses are distinct from the known viral diversity and constitute undescribed taxa. These viruses often had a basal position within their groups suggesting that parasitic flatworms played a role in virus evolution. Previous studies showed that the order *Jingchuvirales* has an ancestral position to the order *Mononegavirales* [54]. SsJV1 has a rather basal position within the order *Jingchuvirales* but the low support of the branch calls for greater sampling of viruses of flatworms. Similarly, SsTV1 and SsTV3 clustered together and had a basal position relative to closely related viruses found in other invertebrates. SsBV1 did not cluster with known viruses and its phylogenetic position fell between the two main clusters of viral families that constitute the order *Bunyavirales*, again indicating a distinct nature from other viruses, and potential ancestral position.

Most strikingly, we found that SsRV1 clusters together with viruses of unidentified hosts and together they constitute a new taxa that shares ancestry with lyssaviruses and other viruses of the dimarhabdoviruses supergroup. The family *Rhabdoviridae* includes viruses of vertebrates, invertebrates, and plants grouped within 20 genera [56–58]. Well-known viruses within this family include viruses of public health, veterinary, and agricultural importance, such as the rabies virus, vesiculoviruses, and potato yellow dwarf virus [59]. The phylogenetic position of SsRV1 and relatives, within the large gap between rhabdoviruses of plants and invertebrates and rhabdoviruses of vertebrates, suggests that an ancestral virus infecting parasitic flatworms could have been involved in the emergence of rhabdoviruses in vertebrates. In support of this, we showed that SsRV1 is transmitted to both copepods and stickleback hosts over the course of infection and is excreted during breeding, which would offer opportunities for such viruses to switch host if the virus gains the ability to overcome host barriers of infections. Clearly, future studies characterizing a greater diversity of viruses of parasitic flatworms and their phylogenetic position relative to the diversity of viruses within their intermediate and definitive hosts has the potential to fill major gaps in our understanding of virus evolution.

### Could tapeworm viruses impact parasite fitness?

Viruses of parasites can either be beneficial for the parasite by increasing infectivity or transmission to the next host, or they can be hyperparasitic and result in parasite hypovirulence that benefits the parasitized host [13]. Both scenarios will have downstream effects on the evolution of the host–parasite interaction [13, 60]. The prevalence of virus(+) parasites in coinfected fish did not differ from what could be expected from a random subsampling of parasites in the population, indicating that all viruses have a low rate of horizontal transmission in fish. We found that SsRV1, SsJV1, SsTV1, SsTV2, and SsTV3 are all vertically transmitted, some of these at high rate. The optimal evolutionary strategy theory predicts that strict vertical transmission should be rare except for viruses that provide fitness advantages [61], but further studies are needed to experimentally assess the impact of viruses on the flatworm fitness.

The rhabdovirus SsRV1 maintained high prevalence in all three tested populations and our results suggest it could be cross-species transmitted to its hosts. SsRV1 was not found in sticklebacks when parasite infection was not successful, but it was present in copepods that resisted infection by *S. solidus*. Further studies are necessary to assess whether copepods serve as vectors of SsRV1, facilitating horizontal transmission between procercoids. SsRV1 was found in the muscle of the body cavity, and in the spleen, liver, and head kidney of all sticklebacks parasitized by at least one SsRV1(+) parasite. However, it was absent from the gut tissue, even though the parasite comes in close contact to the gut serosa during infection. The absence of SsRV1 in stickleback intestines indicates that the virus is excreted while the parasite develops to sexual maturity in the body cavity [62]. The fish liver, spleen, and head kidneys are involved in many biological processes in sticklebacks, such as immune response to infection by *S. solidus*, metabolism, and energy storage [63–69]. The excretion of SsRV1 by *S. solidus* is likely to stimulate the host immune response, but a putative virus replication in hematopoietic organs could prevent it. Clearly, future studies need to assess the ability of SsRV1 to replicate within parasitized host cells, its impact on hosts immune responses, and susceptibility to parasite infection.

In contrast, SsJV1, SsTV1, SsTV2, SsTV3, and SsBV1 had low prevalence in all tested Alaskan populations. The low prevalence of SsJV1 and SsTV1 is particularly surprising given the estimated high success of vertical transmission. This result suggests that at some point during the parasite life cycle, SsJV1(+) and SsTV1(+) parasites are less successful than SsJV1(−) and SsTV1(−) parasites and that these viruses may negatively impact *S. solidus* fitness. Alternatively, the low prevalence of SsJV1 and SsTV1 may result from competition with the highly prevalent SsRV1. Functional experiments to determine the fitness impact of these viruses, alone and in combination, on *S. solidus* and its stickleback host need to be conducted.

### Perspectives

Viruses and parasites alike have significant impacts in health sciences, but virologists and parasitologists have mostly studied them separately. Herein, we discovered vertically transmitted viruses in the cestode *S. solidus* and showed that at least one of these is transmitted to parasitized hosts. Their phylogenetic positions indicate that parasitic flatworms may have played a role in virus evolution and call for a more comprehensive search for viruses of parasitic flatworms. Further studies are necessary to determine the impact of these viruses on parasite fitness and in host–parasite coevolution. Of interest in medical and veterinary sciences, when parasite-associated viruses are responsible for exacerbated forms of parasite infection, new targeted therapies can be developed to reduce symptoms, as exemplified with the *Leishmaniavirus* [70].

## Supplementary information

Supplementary material

## Data Availability

Sequencing data were submitted to NCBI Sequence Read Archives under Bioproject accession number PRJNA576618. Viral genome sequences were submitted to GenBank under accession numbers MN803432-MN803437.
