# Supplementary material for: Characterization of viruses in a tapeworm: phylogenetic position, vertical transmission, and transmission to the parasitized host
Source: ISME J. 2020 Apr 14;14(7):1755–67. doi: 10.1038/s41396-020-0642-2 (PMC7305300; doi:10.1038/s41396-020-0642-2)
Supplement: Supplementary file 1 — Supplementary material [file 41396_2020_642_MOESM1_ESM.pdf]

Supplementary information

Extended Methods

Materials and Methods

Initial sample processing and sequencing for virus detection

Schistocephalus solidus field-collected specimens were screened for the presence of viruses through viral purification and shotgun sequencing. For this purpose, *S. solidus* plerocercoids were dissected out of four sticklebacks collected in Cheney Lake, Alaska (61° 12' 17" N, 149° 45' 33") in June 2016 resulting in four parasite samples. The plerocercoids were cut into pieces and immediately transferred into phosphate-buffered saline (PBS) for virus purification through filtration followed by chloroform and nuclease treatment according to Ng et al (1, 2) with some modifications to the protocol. Briefly, tissue samples were homogenized in sterile PBS by bead beating with 3mm glass beads. The homogenates were centrifuged for 1min at 6000rpm and pellets were discarded. The recovered supernatants were further diluted with 500µl of PBS and centrifuged at 6000rpm for 6min to remove remaining cell debris. The supernatants were then filtered successively through 0.4µm and 0.22µm sterile cellulose acetate filters (Corning CAT# 8162) and filtrates containing the viral fraction were incubated for 10min in 0.2 volumes of chloroform. The viral fraction was recovered from the aqueous phase after centrifugation for 20s at 20,000rpm. A second chloroform treatment was applied to ensure removal of bacterial contaminants. The viral fraction was further purified by treating with 2.5U of DNase I and 0.25U of RNaseA at 37°C for 3h to eliminate non-encapsidated DNA and RNA. EDTA (pH = 8, Sigma Aldrich CAT# E7889) was added at a final concentration of 20mM to inactive nucleases prior to nucleic acid extraction.

Nucleic acids were extracted using the QIAamp Mini Elute Virus Spin Kit according to manufacturer's instructions and DNA was removed using a Turbo DNAse treatment (Thermofisher CAT# AM1907). Four sequencing libraries, one per parasite sample, were prepared with the NuGen Oviation Universal RNA-Seq System (CAT#0343) following the standard protocol and 18 PCR cycles. Libraries were used for single-end sequencing (1 x 150bp) on an Illumina Hi-Seq 4000 (Institute of Biotechnology at Cornell University). We obtained 10.22, 12.57, 10.81 and 1.54 Mi reads for each respective sample. Sequences were processed through Stony Brook University Seawulf high performance computing cluster. For each dataset, adapters

were removed using Trimmomatic version 0.36 with default settings and PhiX174 contaminants were removed using Bowtie 2 (--very-sensitive-local) (3). Sequence quality after trimming was verified with FastQC version 0.11.5 (4). D*e novo* assembly was completed by pooling sequence data from all four samples using Trinity (31). Contigs representing partial viral sequences similar to various rhabdoviruses and chuviruses were identified through BLAST searches against GenBank non-redundant database (BLASTx, e-value < 10^{-10}). The partial sequences represented two viruses, a rhabdovirus, named Schistocephalus solidus Rhabdovirus (SsRV1) and a chuvirus named Schistocephalus solidus Jingchuvirus (SsJV1).

Sampling and *in-vitro* culturing of *S. solidus* for virus genome sequencing and experimental infections

Field sampling

The detection of viral sequences in plerocercoids collected from Cheney Lake prompted further sampling of *S. solidus* from various lakes to screen for other viruses, evaluate the prevalence and distribution of detected viruses, and perform experimental infections. In June of 2018, 31, 20, and 46 plerocercoids were collected from sticklebacks fished from Cheney Lake, Wolf Lake (61° 38' 36" N, 149° 16' 32" W), and Loberg Lake (61° 33' 33.5" N 149° 15' 28.9" W), respectively. For a subset of stickleback hosts, the liver was collected. Whole plerocercoids and fish tissue samples were transferred into RNA later for future analyses.

***In-vitro culture of S. solidus plerocercoid*s**

Freshly collected plerocercoids were used for in vitro breeding by placing size-matched pairs into sealed biopsy bags (5-7). Each pair was incubated for 4 days at 40°C into 250ml of Minimum Essential medium (MEM Sigma M2279) enriched with HEPES buffer (Sigma CAT# 83264, 50ml l^{-1}), Antibiotic antimycotic (Sigma CAT# A5955, 10ml l^{-1}), L-glutamin (Sigma CAT# G7513, 10mmol l^{-1}) and glucose (Sigma CAT# G7021, 40ml l^{-1}). Every 48h, eggs were collected and the culture medium was replaced. Eggs were washed 5 times in sterile water and stored at 4°C in the dark for less than a year. We obtained eggs from16 families from Cheney lake, 13 families from Wolf lake, and 9 families from Loberg lake, where a family refers to eggs collected from the breeding of one parasite pair. Parent parasites used for breeding were transferred into RNA later and a sample of culture medium was mixed V/V with RNA later for future analyses.

Egg hatching

To stimulate egg hatching, eggs were incubated in deionized water for 3 weeks at 18-22°C in the dark before being exposed to UV light for 1h, placed in the dark overnight and exposed to UV light for 3 more hours (5). Newly hatched coracidia were collected through centrifugation at 6,500 rpm or 5 min at 4°C and either processed for experimental infections, virus purification, or directly transferred in RNA later for future analyses.

Virus detection and sequencing

Newly hatched coracidia from *S. solidus* families from Wolf Lake (five families), Loberg Lake (two families), and Cheney Lake (two families) were used for a second sequencing effort to complete the genomes of SsRV1 and SsJV1 and potentially detect more viruses (Table S1). To purify viruses, coracidia were homogenized in sterile suspension medium (SM) buffer [100 mM NaCl, 8 mM $MgSO_4 \cdot 7H2O$, 50 mM Tris-Cl (pH = 7.5)] through bead beating in a Fisherbrand Bead Mill 4 homogenizer (Fisher Scientific CAT# 15-340-164) for 1min using a mixture of 0.1mm and 1mm glass beads. Homogenates were then centrifuged at 8,000 x g for 10min and the supernatants containing the viral fraction were filtered through a 0.45µm Sterivex filter (Fischer Scientific CAT# SVHV010RS) to remove cells. Free DNA and RNA were removed from the viral fraction by incubating filtrates with a nuclease cocktail consisting of 1X Turbo DNase Buffer, 21U of Turbo DNase (Fisher Scientific CAT# AM2238), 4.5U of Baseline-ZERO DNase (Epicenter CAT# DB0711K), 112.5U Benzonase (Fisher Scientific CAT# 707463), and 10 µg/mL RNase A (Fisher Scientific CAT# AM2294) at 37 °C for 2 h. Nucleases were inactivated with 20 mM EDTA prior to nucleic acid extraction.

Viral RNA was extracted from 200µl of purified viral fraction using the RNeasy kit (Qiagen CAT# 74104) with the on-column DNase digestion step following manufacturer's recommendations. In addition, total RNA extracts obtained from plerocercoids and coracidia from each lake (see below) were processed alongside RNA extracts from the purified viral fraction. Note that both viral and total RNA extracts processed for this second sequencing effort came from parasite families known be positive for SsRV1 and/or SsJV1 (Table S1). RNA was reverse-transcribed using the SuperScript IV First Strand Synthesis System (Fisher Scientific CAT#18091050) with random hexamers followed by second-strand cDNA synthesis using the Klenow Fragment DNA polymerase (New England Biolabs CAT#M0212S). The resulting products were cleaned using the ZR DNA Clean & Concentrator kit -25 (Zymo ResearcgZ CAT#

D4006). Purified cDNA samples from the viral fraction (V) and those from total RNA (T) were pooled into two samples, namely the V-pool and the T-pool. Details regarding the number of families and subsamples that were included in each pool are provided in Table S1. Both pools were fragmented to 300 bp using a Covaris M220 instrument at the Molecular Genomics Core at the H. Lee Moffitt Cancer Center & Research Institute. Next-generation sequencing library construction was performed with the Accel-NGS 1S Plus DNA Library Kit for Illumina Platforms (Swift Biosciences CAT# 10024) following manufacturer's instructions for DNA inputs <1 ng/µl and 18 cycles of dual indexing PCR for the V-library. For the T-library, fragmented RNA was processed following the protocol for DNA inputs > 10 ng/ul and 10 cycles of dual indexing PCR. Both libraries were commercially paired-end sequenced (2 × 150 bp) on an Illumina HiSeq 4000 System at GENEWIZ.

Sequences were processed through the University of South Florida high performance computing cluster. Raw sequences were trimmed for quality and to remove indexing adapters using Trimmomatic version 0.36.0 (8) with default parameters except for a read head crop of 10 bp instead of zero. Sequence quality after trimming was verified with FastQC version 0.11.5 (4). Due to the high number of indexing PCR cycles, quality-filtered sequences from the V-library were assembled following a pipeline for PCR amplified libraries (9). To do this, sequences were dereplicated using the Clumpify tool from the BBtools package (sourceforge.net/projects/bbmap/). Dereplicated sequences were then assembled using single cell SPAdes (10). Quality-filtered sequences from the T-library were assembled with RNAspades. Contigs larger than 1000 bp were compared (BLASTx, e-value < 10^{-10}) against a viral protein database containing sequences from the NCBI Reference Sequence database (RefSeq Release number 93, https://www.ncbi.nlm.nih.gov/refseq/). This sequencing effort resulted in the detection of contig sequences representing SsJV1, SsRV1, SsBV1 and three novel toti-like viruses, named SsTV1, SsTV2 and SsTV3.

Viral genome completion

Quality filtered reads and contig sequences associated with each of the viruses were retrieved by comparing sequences through BLASTn to a database containing newly identified contig sequences, including those from the original assembly done in Trinity, and closely-related sequences. All reads and contigs were re-assembled using the default overlap-consensus

algorithm implemented in Geneious version R7. All assemblies resulted in near-complete genome sequences represented by a single contig, with the exception of SsRV1 for which genome gaps were closed through targeted PCR (primers listed on Table S2). To complete genomes, RNA samples from families originally pooled by lake were screened for each of the viruses to identify positive samples. Positive samples were then used for PCR and rapid amplification of complimentary ends (RACE) assays (11). PCRs were performed using the AmpliTaq Gold™ 360 Master Mix with GC enhancer (Thermo Fisher Scientific). PCR using primers designed off of the SsJV1 contig sequence ends confirmed the circular topology of this new chuvirus-like genome (Table S2). The genome ends of the remaining viral sequences were completed through RACE (primers provided in Table S3). Prior to 3'RACE reactions, RNA extracts were denatured at 95ºC for 6 minutes and placed on ice immediately to prevent RNA reannealing (12). Denatured RNA was used as template for poly(A) tail reactions using a Poly(A) Polymerase from *E. coli,* which synthetizes poly(A) tails at the 3' termini of ssRNA templates. Poly(A) reactions contained 1mM ATP, 1X poly(A) polymerase buffer, 0.25 U poly(A) polymerase (New England Biolabs) and 15 ul of RNA. Poly(A)-tailed RNAs were used as template for 3'RACE reactions. The 5' ends were completed with the 5'RACE system. All RACE products were cloned using the CloneJET PCR Cloning Kit (Thermo Fisher Scientific) and Sanger sequenced using vector primers. All PCR and cloned RACE products were commercially sequenced by TACGen.

Virus genome characterization and phylogenetic analysis

The prediction of open reading frame was obtained using Translate on ExPASy and from alignments with related reference virus genomes. Annotation of domains was extracted from comparisons against the Conserved Domain Database (CDD) as implemented by Blastp against the non-redundant protein database. Initial supergroup assignation was determined from best blast hit. The viral RdRP were aligned using the E-INS-I algorithm implemented in the program MAFFT (version 7) (13) to representative sequences of all families and genus of viruses ratified by the ICTV, as well as additional newly described taxa from recent meta-transcriptomic studies (14-17). Next, all ambiguously aligned regions were removed using TrimAl (version 1.2) (18). For each dataset, the best-fit model of amino acid substitution was determined using Smart Model Selection (SMS) (19). Phylogenetic tree were then inferred using the maximum likelihood

method implemented in PhyML (version 3.0) (19) using the best-fit model and best of NNI and Subtree Pruning and Regrafting (SPR) branch swapping. Support for nodes on the trees were assessed using an approximate likelihood ratio test (aLRT) with the Shimodaira-Hasegawa-like procedure.

Experimental infections of copepods

A highly susceptible strain of *Macrocyclops albidus* copepods from lake Skogseidsvatnet, Norway (20) were cultured in the laboratory at 20°C and 16:8 light:dark cycle. C5 copepodite stage were exposed to one coracidium each as previously described (20). Fourteen days post exposure, copepods were either directly collected and stored in RNA later, or screened under the microscope to determine the infection success. Sham-exposed, infected and non-infected copepods were then individually sorted, rinsed in sterile water, isolated via centrifugation and transferred in RNAlater. To control for potential contamination by *S. solidus* in exposed but non-infected copepods, we conducted PCRs with *S. solidus* specific primers according to Berger et al (21).

Sampling and experimental infection of threespine sticklebacks

In June 2018, we collected mature males and gravid females of threespine sticklebacks, *Gasterosteus aculeatus,* from Rabbit Slough (61° 32' 08.1" N 149° 15' 10.0" W), Cheney Lake, and Loberg lake and completed crosses *in vitro* to obtain lab-bred families. Fish were reared in the laboratory at 18°C and 16:8 light:dark cycle until they were 5-months old and ready for experimental exposure to *S. solidus*. Fish were fed a diet of frozen brine shrimps and chironomids larvae ad libitum daily. A total of 224 fish were exposed to copepods parasitized either by virus-free *S. solidus* (45 individuals), by SsRV1(+)/SsJV1(+) *S. solidus* (46 individuals), by SsRV1(-)/SsJV1(+) *S. solidus* (46 individuals), or by SsRV1(+)/SsJV1(-) *S. solidus* (72 individuals) with a single *S. solidus* infected with SsRV1, SsJV1, or neither virus. Exposure was performed by placing a single infected copepod in a tank containing a single fish that had been starved for 48h. Two days later, fish were transferred back into large tanks. After eight weeks, fish were dissected and when infected, plerocercoids, fish body cavity, liver and intestine were collected and stored in RNA later.

PCR assays for assessing viral prevalence and transmission

Specimens collected at different stages of *S. solidus* life cycle were used to assess virus presence. Total RNA was extracted from culture medium used for breeding, coracidia, copepods, and

stickleback tissues using the RNeasy kit (Qiagen CAT#74106) with the on-column DNase digestion step following the manufacturer's guidelines. Total RNA from plerocercoids was extracted using Trizol^{TM} reagent (Fisher Scientific CAT#15-596-018) following the manufacturer's recommendations before DNase treatment with Turbo DNA free kit (Ambion® CAT#AM1907). First strand cDNA was synthesized by reverse transcribing 500ng of tRNA with Random Hexamer Primer and RevertAid H Minus Reverse Transcriptase (Thermo Fisher Scientific CAT# EP0451), as per manufacturer's recommendations. Polymerase chain reaction was conducted using the Advantage 2 PCR system (Invitrogen CAT# 639137) using primers targeting the conserved RdRP gene of viruses (Table S2). Amplicon presence was assayed on 1% agarose gel with SyBR Safe. Select PCR products were sequenced using Sanger sequencing to confirm primers' specificity.

Data mining

To assess virus presence in other populations of *S. solidus*, we queried BioProjects of publicly available transcriptomes. BLASTn searches were used to determine the presence of 454 reads that aligned to the newly identified viruses in data from PRJEB7355 (https://www.ncbi.nlm.nih.gov/bioproject/316954, 2 biosamples of wild-caught Norwegian and German *S. solidus*) and PRJNA304161 (15 biosamples from Clatworthy reservoir, England, UK (22)). Sequence data from PRJNA304161 were then downloaded and processed as follows: reads were trimmed with the Trimmomatic version 0.36 (8) with default settings. Quality filtered reads were aligned against the *S. solidus* reference genome (GCA_900618435.1) with Bowtie2 (version 2.3.4.1) (3). Unmapped reads were collected using SAMtools 1.8 (23) and bedtools (24) and assembled using the shovill method (https://github.com/tseemann/shovill).To identify viral contigs, we used BLASTx against the nr database as described above. To test for virus presence in all individuals and provide relative quantitation, clean reads were aligned on assembled viruses using BWA (version 0.7.8) (25).

Ethics statement

Stickleback collection followed guidelines for scientific fish collection by the State of Alaska Department of Fish and Game in accordance with Fish sampling permit #P17-025 and #P-18-008 and fish transport permits 17A-0024. Fish were maintained at Stony Brook University under the License to collect or possess #1949 provided by the New York State Department of Environmental

Conservation and experiments were conducted following protocols described in Institutional Animal Care and Use Committee (IACUC) #237429 and # 815164. All experiments were performed in accordance with relevant guidelines and regulations in the Public Health Service Policy (PHS) on Humane Care and Use of Laboratory Animals.

Table S1. Details regarding the samples used for the viral (V) and total RNA (T) NGS library preparations, including the origin and number of parental parasite families represented in a given sample, the *Schistocephalus solidus* life stage, and if parental families were positive for the S. solidus rhabdovirus (SsRV1), S. solidus jingchuvirus (SsJV1) or both. Each sample was extracted and reverse-transcribed followed by second-strand cDNA synthesis. Double-stranded cDNA samples were then combined into a single sample representing either the V- or T-pool for cleanup and library preparation.

Library	Lake (# of families)	Life stage	SsRV1 and SsJV1	SsRV1	SsJV1
V	Wolf (2)	Coracidia	X		
V	Wolf (1)	Coracidia			X
V	Wolf (2)	Coracidia		X	
V	Loberg (2)	Coracidia		X	
V	Cheney (2)	Coracidia		X	
T	Wolf (3)	Plerocercoids and coracidia	X		
T	Cheney (2)	Plerocercoids and coracidia	X		
T	Loberg (1)	Plerocercoids	X		
T	Loberg (2)	Plerocercoids		X	
T	Cheney (5)	Coracidia		X	
T**	Cheney	Plerocercoids	X		

**This sample included RNA extracted from purified viral particles

Table S2. Primer pairs used to detect viruses in *Schistocephalus solidus* samples.

*Primer name specifies the targeted virus. SsRV1, Schistocephalus solidus rhabdovirus; SsJV1, Schistocephalus solidus jingchuvirus; SsBV1, Schistocephalus solidus bunya-like virus; SsTV1, Schistocephalus solidus toti-like virus 1; SsTV2, Schistocephalus solidus toti-like virus 2; SsTV3, Schistocephalus solidus toti-like virus 3.

**Primer ID**	**Sequence (5' – 3")**	**Product Length (bp)**	**Annealing Temp (ºC)**	**Purpose**
SsRV1_F3<br>SsRV1_R3	CCGTTAAGGCCGATGTTTTA<br>AGTTGACTACGCCCCAGTTG	100	57	Screening
SsRV1_F4<br>SsRV1_R4	TTGTCAACTGGGGCGTAGTC<br>TCGTTACGGAAGGAGGAGGT	386	57	Screening
SsRV1_F5<br>SsRV1_R5	ACCTTGTGTGGCTCGATGAT<br>GGCTGAAAATGGAAAACGAG	500	52	Bridge genome gap
SsRV1_F6<br>SsRV1_R6	TGTGTCATTCAGGGTTTCCA<br>AGTTTGGACAGACCGCATTC	700	52	Bridge genome gap
SsRV1_F7<br>SsRV1_R7	GCATCCTCCCTCCATCATAA<br>ACTGCAAAGTCCCAACAACC	550	52	Bridge genome gap
SsJV1_F3<br>SsJV1_R3	TCGTCTTCCCGTAAACGAAC<br>ATTCGTACCGGACAGCACTC	425	57	Screening
SsJV1_F4<br>SsJV1_R4	CGCTTTACCACCTTCCCTGT<br>CTTGGCGTCCGTTTCCTAGT	280	57	Screening
SsJV1_F5<br>SsJV1_R5	TGGTGTGTGGTGTTTGGTCT<br>CCCTCGGGTAGTTCAAAGGA	300	57	Verify circular nature
SsBV1_F1<br>SsBV1_R1	ATCATGCAGTGGACCAAGGA<br>ATGGTGTCCCTCTTGAGGTG	900	55	Screening
SsTV1_F1<br>SsTV1_R1	CTCCTATACCGGTCCCCAAC<br>GCTGATAACCGCCAGAGTTC	690	55	Screening
SsTV2_F1<br>SsTV2_R1	TTGGCTTTTACCAGGGTTTG<br>AAATCCAGCGTCTGACAACC	620	52	Screening
SsTV3_F2<br>SsTV3_R2	CTGGAGGGGCTTAGTCTCTG<br>CAAAGCCGGAGTGATCGAAG	970	55	Screening

Table S3. Gene specific primers (GSP) used for rapid amplification of complimentary ends (RACE) assays.

**Primer ID**	**Sequence (5' – 3")**	**End**
SsRV1_GSP1	ATGTTGGCCATCTCTTTGCT	3'
SsRV1_GSP2	TCAGGAACACCTGCGTTACA	3'
SsRV1_GSP3	TGGAGAGATCGGGCAATTTA	3'
SsRV1_GSP4	CAAAGTGCCCTGGTTGTTCT	5'
SsRV1_GSP5	TGGATATAGGCGCTACATTGG	5'
SsRV1_GSP6	AGTGTTGGACTCATTGCGTC	5'
SsBV1_GSP1	GGAGAGGGAGATCGTCAACA	3'
SsBV1_GSP2	GTGGAACAGCAAACACTGGA	3'
SsBV1_GSP3	CATCACCGAGAACTTCACGA	3'
SsBV1_GSP4	GGCAAACATCACCTCCTTGT	5'
SsBV1_GSP5	AGGGCATGTTGTTGATGACA	5'
SsBV1_GSP6	ACCACCAGCAAGGTCTTCAC	5'
SsTV1_GSP1	TCCTCAAGTCCCTGAACCAG	3'
SsTV1_GSP2	TTACGTGGACTGAGGGCATT	3'
SsTV1_GSP3	CACCAAAAATGTATTCGCCC	3'
SsTV1_GSP4	GAGAAGCTGTCCACAGACG	3'
SsTV1_GSP5	CAGTTTCTGCGTCACCCTTT	3'
SsTV1_GSP6	TGGGCTCTCACTGTATTCGC	3'
SsTV2_GSP1	AGTATGTCCCCGATGTGGAG	3'
SsTV2_GSP2	CTGAGGGGACTGACTGGTGT	3'
SsTV2_GSP3	GCTTTCCTCACAGGAACTGG	3'
SsTV2_GSP4	GGACGAATGGATTGGAGATG	3'
SsTV3_GSP1	CCACCCTTTCATCTGCCTAA	3'
SsTV3_GSP2	ATGATAGGGGTGGCAGAGTG	3'
SsTV3_GSP3	GTTTGAAGCCATGGGAGAAC	3'
SsTV3_GSP4	ACCTGGCAGAGGCAATTAGA	3'
SsTV3_GSP5	AAAGATGAATGGGTCGGTGA	3'
SsTV3_GSP6	TCTATTCGGGCCTACAGGAG	3'

Table S4: transcription initiation and termination signals in SsRV1 genome

ORF	Intergenic	Transcription initiation sequence	Termination signal
N		**UUGU**UGUGUAUAUUUGCUUUUAACAGUGAACGCCCCAUAC	UCUUUUUUU
P	G	**UUGU**UAC	UCUUUUUUU
M	G	**UUGU**UUUUUCCUUUUUGGCUCUGUAGUAC	UCUUUUUUU
G	G	**UUGU**GUUGUGGAACUUGGGAUGUCGUUCUUGGUCUUAAGGUAACGCUUGCACUCUUAC	UCUUUUUUU
α1	G	**UUGU**AGAUUGACGGGGCCCCUCCUGUGUGUAC	UCUUUUUUU
L	G	**UUGU**UAGUAAACCAUUCGAUCUGUUUAACAAUGACACUAACUAGUAC	UCUUUUUUU

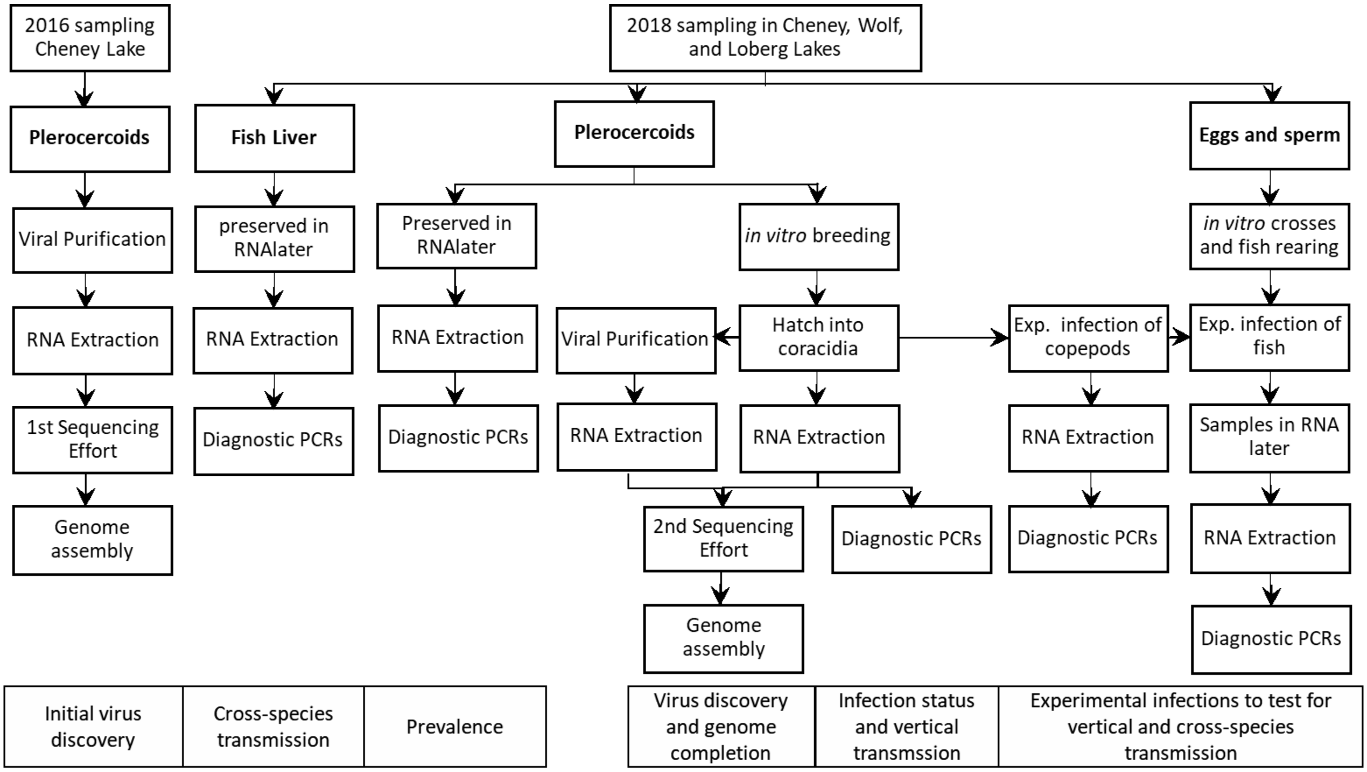

Figure S1: Overview of sampling effort and processing steps taken during this study to investigate *Schistocephalus solidus* viruses.

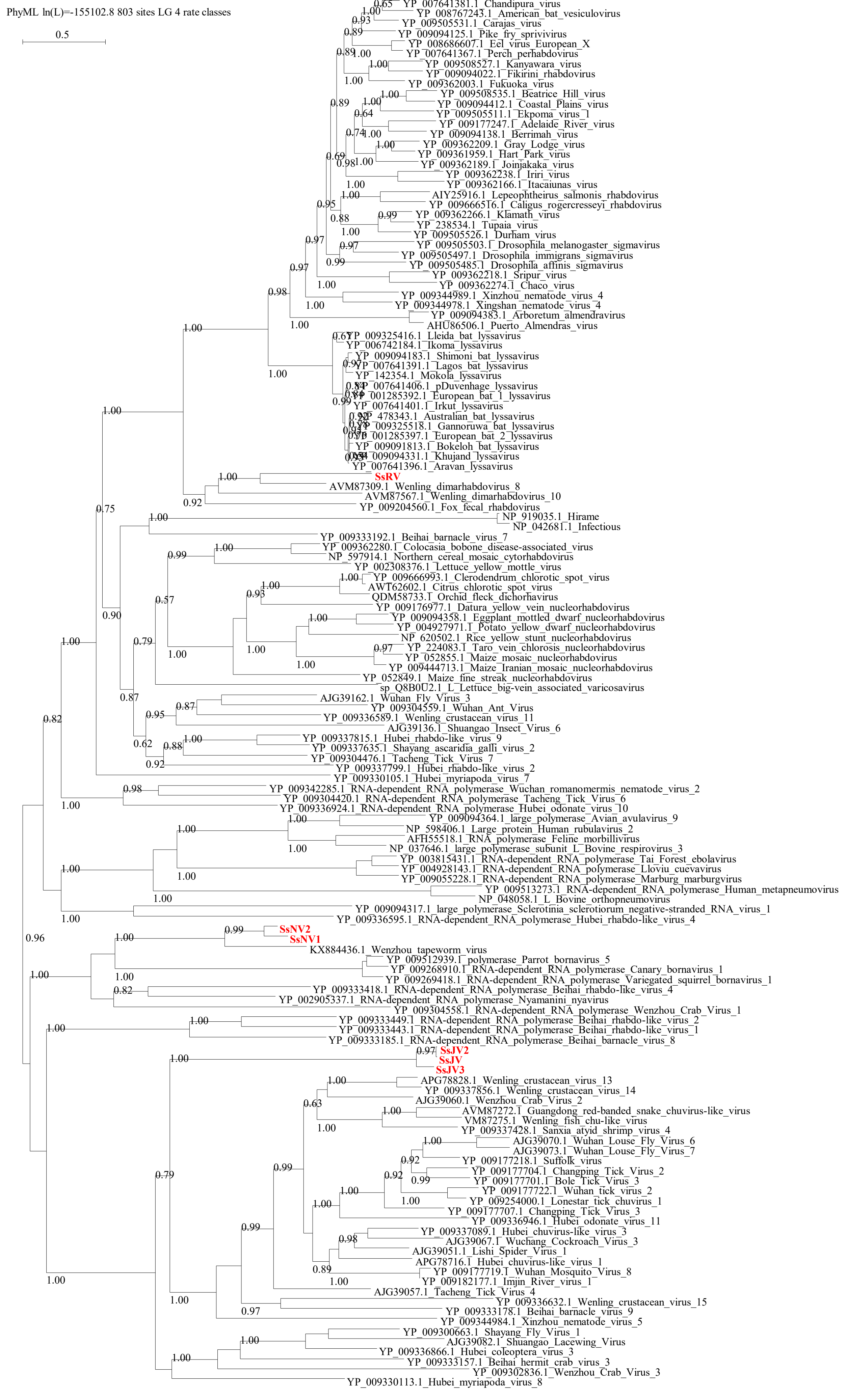
PhyML ln(L)=-155102.8 803 sites LG 4 rate classes
0.5
YP_007641381.1_Chandipura_virus
YP_008767243.1_American_bat_vesiculovirus
YP_009505531.1_Carajas_virus
YP_009094125.1_Pike_fry_sprivivirus
YP_008686607.1_Eel_virus_European_X
YP_007641367.1_Perch_perhabdovirus
YP_009508527.1_Kanyawara_virus
YP_009094022.1_Fikirini_rhabdovirus
YP_009362003.1_Fukuoka_virus
YP_009508535.1_Beatrice_Hill_virus
YP_009094412.1_Coastal_Plains_virus
YP_009505511.1_Ekpoma_virus_1
YP_009177247.1_Adelaide_River_virus
YP_009094138.1_Berrimah_virus
YP_009362209.1_Gray_Lodge_virus
YP_009361959.1_Hart_Park_virus
YP_009362189.1_Joinjakaka_virus
YP_009362238.1_Iriri_virus
YP_009362166.1_Itacaiunas_virus
AIY25916.1_Lepeophtheirus_salmonis_rhabdovirus
YP_009666516.1_Caligus_rogercresseyi_rhabdovirus
YP_009362266.1_Klamath_virus
YP_238534.1_Tupaia_virus
YP_009505526.1_Durham_virus
YP_009505503.1_Drosophila_melanogaster_sigmavirus
YP_009505497.1_Drosophila_immigrans_sigmavirus
YP_009505485.1_Drosophila_affinis_sigmavirus
YP_009362218.1_Sripur_virus
YP_009362274.1_Chaco_virus
YP_009344989.1_Xinzhou_nematode_virus_4
YP_009344978.1_Xingshan_nematode_virus_4
YP_009094383.1_Arboretum_almendravirus
AHU86506.1_Puerto_Almendras_virus
YP_009325416.1_Lleida_bat_lyssavirus
YP_006742184.1_Ikoma_lyssavirus
YP_009094183.1_Shimoni_bat_lyssavirus
YP_007641391.1_Lagos_bat_lyssavirus
YP_142354.1_Mokola_lyssavirus
YP_007641406.1_pDuvenhage_lyssavirus
YP_001285392.1_European_bat_1_lyssavirus
YP_007641401.1_Irkut_lyssavirus
YP_478343.1_Australian_bat_lyssavirus
YP_009325518.1_Gannoruwa_bat_lyssavirus
YP_001285397.1_European_bat_2_lyssavirus
YP_009091813.1_Bokeloh_bat_lyssavirus
YP_009094331.1_Khujand_lyssavirus
YP_007641396.1_Aravan_lyssavirus
SsRV
AVM87309.1_Wenling_dimarhabdovirus_8
AVM87567.1_Wenling_dimarhabdovirus_10
YP_009204560.1_Fox_fecal_rhabdovirus
NP_919035.1_Hirame
NP_042681.1_Infectious
YP_009333192.1_Beihai_barnacle_virus_7
YP_009362280.1_Colocasia_bobone_disease-associated_virus
NP_597914.1_Northern_cereal_mosaic_cytorhabdovirus
YP_002308376.1_Lettuce_yellow_mottle_virus
YP_009666993.1_Clerodendrum_chlorotic_spot_virus
AWT62602.1_Citrus_chlorotic_spot_virus
QDM58733.1_Orchid_fleck_dichorhavirus
YP_009176977.1_Datura_yellow_vein_nucleorhabdovirus
YP_009094358.1_Eggplant_mottled_dwarf_nucleorhabdovirus
YP_004927971.1_Potato_yellow_dwarf_nucleorhabdovirus
NP_620502.1_Rice_yellow_stunt_nucleorhabdovirus
YP_224083.1_Taro_vein_chlorosis_nucleorhabdovirus
YP_052855.1_Maize_mosaic_nucleorhabdovirus
YP_009444713.1_Maize_Iranian_mosaic_nucleorhabdovirus
YP_052849.1_Maize_fine_streak_nucleorhabdovirus
sp_Q8B0U2.1_L_Lettuce_big-vein_associated_varicosavirus
AJG39162.1_Wuhan_Fly_Virus_3
YP_009304559.1_Wuhan_Ant_Virus
YP_009336589.1_Wenling_crustacean_virus_11
AJG39136.1_Shuangao_Insect_Virus_6
YP_009337815.1_Hubei_rhabdo-like_virus_9
YP_009337635.1_Shayang_ascaridia_galli_virus_2
YP_009304476.1_Tacheng_Tick_Virus_7
YP_009337799.1_Hubei_rhabdo-like_virus_2
YP_009330105.1_Hubei_myriapoda_virus_7
YP_009342285.1_RNA-dependent_RNA_polymerase_Wuchan_romanomermis_nematode_virus_2
YP_009304420.1_RNA-dependent_RNA_polymerase_Tacheng_Tick_Virus_6
YP_009336924.1_RNA-dependent_RNA_polymerase_Hubei_odonate_virus_10
YP_009094364.1_large_polymerase_Avian_avulavirus_9
NP_598406.1_Large_protein_Human_rubulavirus_2
AFH55518.1_RNA_polymerase_Feline_morbillivirus
NP_037646.1_large_polymerase_subunit_L_Bovine_respirovirus_3
YP_003815431.1_RNA-dependent_RNA_polymerase_Tai_Forest_ebolavirus
YP_004928143.1_RNA-dependent_RNA_polymerase_Lloviu_cuevavirus
YP_009055228.1_RNA-dependent_RNA_polymerase_Marburg_marburgvirus
YP_009513273.1_RNA-dependent_RNA_polymerase_Human_metapneumovirus
NP_048058.1_L_Bovine_orthopneumovirus
YP_009094317.1_large_polymerase_Sclerotinia_sclerotiorum_negative-stranded_RNA_virus_1
YP_009336595.1_RNA-dependent_RNA_polymerase_Hubei_rhabdo-like_virus_4
SsNV2
SsNV1
KX884436.1_Wenzhou_tapeworm_virus
YP_009512939.1_polymerase_Parrot_bornavirus_5
YP_009268910.1_RNA-dependent_RNA_polymerase_Canary_bornavirus_1
YP_009269418.1_RNA-dependent_RNA_polymerase_Variegated_squirrel_bornavirus_1
YP_009333418.1_RNA-dependent_RNA_polymerase_Beihai_rhabdo-like_virus_4
YP_002905337.1_RNA-dependent_RNA_polymerase_Nyamanini_nyavirus
YP_009304558.1_RNA-dependent_RNA_polymerase_Wenzhou_Crab_Virus_1
YP_009333449.1_RNA-dependent_RNA_polymerase_Beihai_rhabdo-like_virus_2
YP_009333443.1_RNA-dependent_RNA_polymerase_Beihai_rhabdo-like_virus_1
YP_009333185.1_RNA-dependent_RNA_polymerase_Beihai_barnacle_virus_8
SsJV2
SsJV
SsJV3
APG78828.1_Wenling_crustacean_virus_13
YP_009337856.1_Wenling_crustacean_virus_14
AJG39060.1_Wenzhou_Crab_Virus_2
AVM87272.1_Guangdong_red-banded_snake_chuvirus-like_virus
VM87275.1_Wenling_fish_chu-like_virus
YP_009337428.1_Sanxia_atyid_shrimp_virus_4
AJG39070.1_Wuhan_Louse_Fly_Virus_6
AJG39073.1_Wuhan_Louse_Fly_Virus_7
YP_009177218.1_Suffolk_virus
YP_009177704.1_Changping_Tick_Virus_2
YP_009177701.1_Bole_Tick_Virus_3
YP_009177722.1_Wuhan_tick_virus_2
YP_009254000.1_Lonestar_tick_chuvirus_1
YP_009177707.1_Changping_Tick_Virus_3
YP_009336946.1_Hubei_odonate_virus_11
YP_009337089.1_Hubei_chuvirus-like_virus_3
AJG39067.1_Wuchang_Cockroach_Virus_3
AJG39051.1_Lishi_Spider_Virus_1
APG78716.1_Hubei_chuvirus-like_virus_1
YP_009177719.1_Wuhan_Mosquito_Virus_8
YP_009182177.1_Imjin_River_virus_1
AJG39057.1_Tacheng_Tick_Virus_4
YP_009336632.1_Wenling_crustacean_virus_15
YP_009333178.1_Beihai_barnacle_virus_9
YP_009344984.1_Xinzhou_nematode_virus_5
YP_009300663.1_Shayang_Fly_Virus_1
AJG39082.1_Shuangao_Lacewing_Virus
YP_009336866.1_Hubei_coleoptera_virus_3
YP_009333157.1_Beihai_hermit_crab_virus_3
YP_009302836.1_Wenzhou_Crab_Virus_3
YP_009330113.1_Hubei_myriapoda_virus_8

Figure S2: Phylogenetic analysis of the RdRP of viruses from the orders *Mononegavirales* and *Jingchuvirales*.

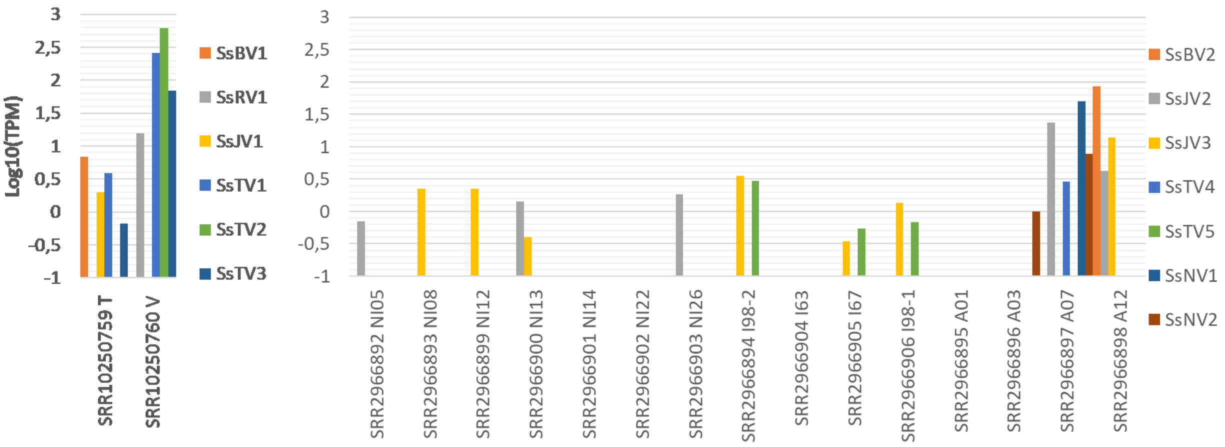

Figure S3: Virus abundance in samples. The figure shows the log of the number of transcripts per millions of clean reads (TPM). NI: non-infective plerocercoids; I: infective plerocercoids; A: adults.

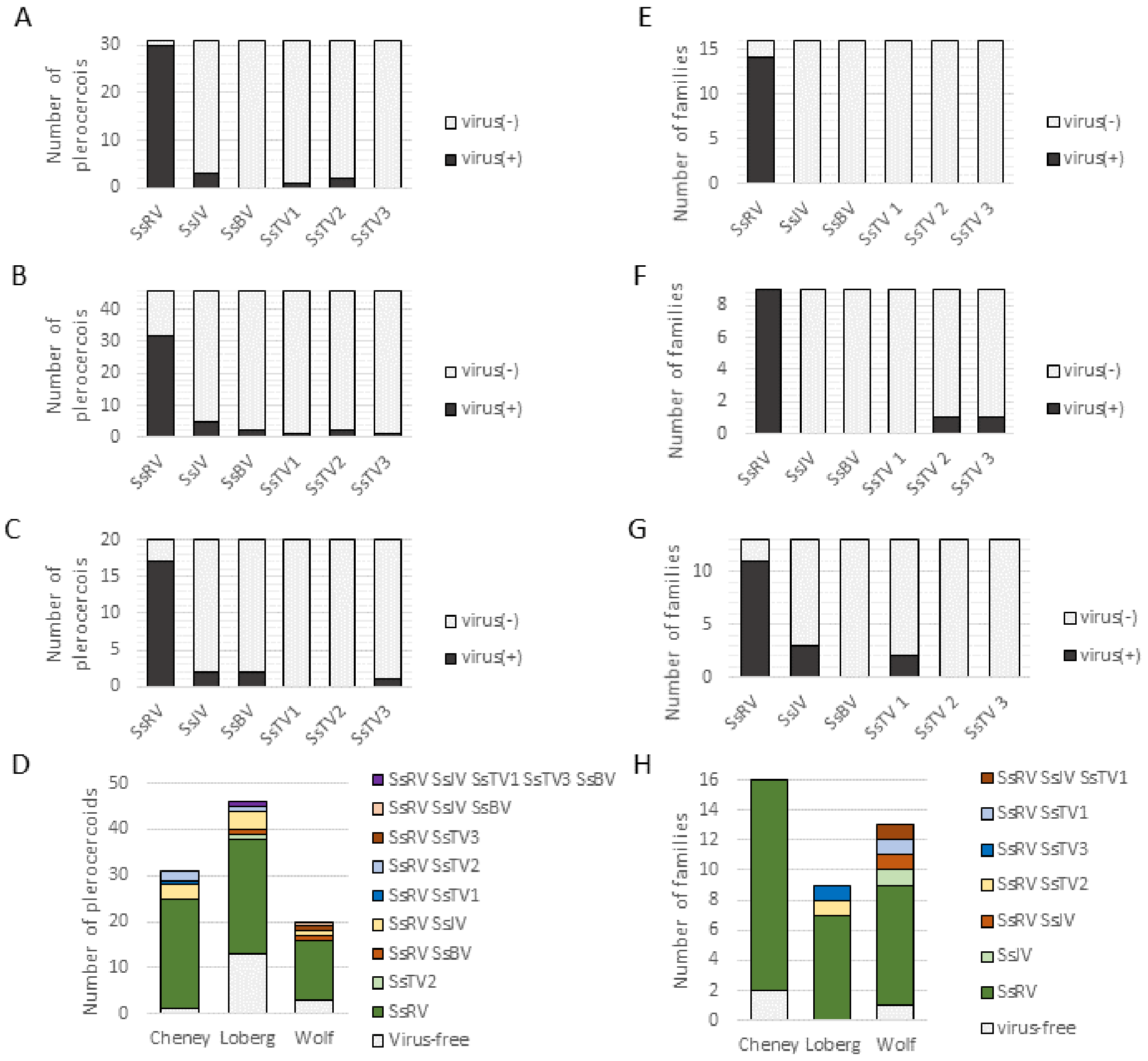

Figure S4: Virus prevalence in plerocercoids (A-D) and presence in families from *in vitro* breeding (E-F). Virus presence in Cheney lake (A and E), Loberg Lake (B and F), and Wolf Lake (C and G). Plerocercoids and families were often coinfected by multiple viruses (D and H).

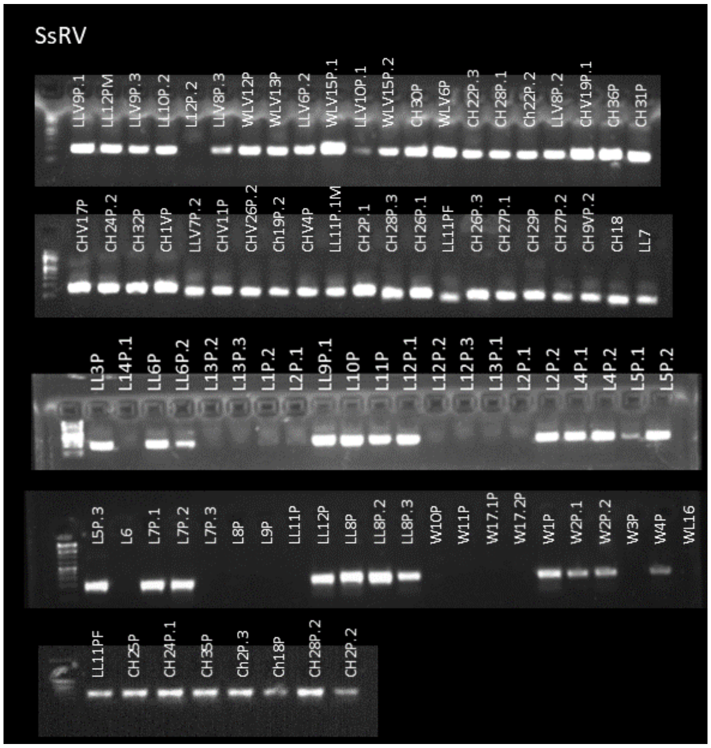

Figure S5: Detection of SsRV1 in plerocercoids from Cheney Lake (CL, 31 individuals), Loberg Lake (LL, 46 individuals) and Wolf lake (WL, 20 individuals). .1 .2 and .3 is used to label plerocercoids collected from the same stickleback host.

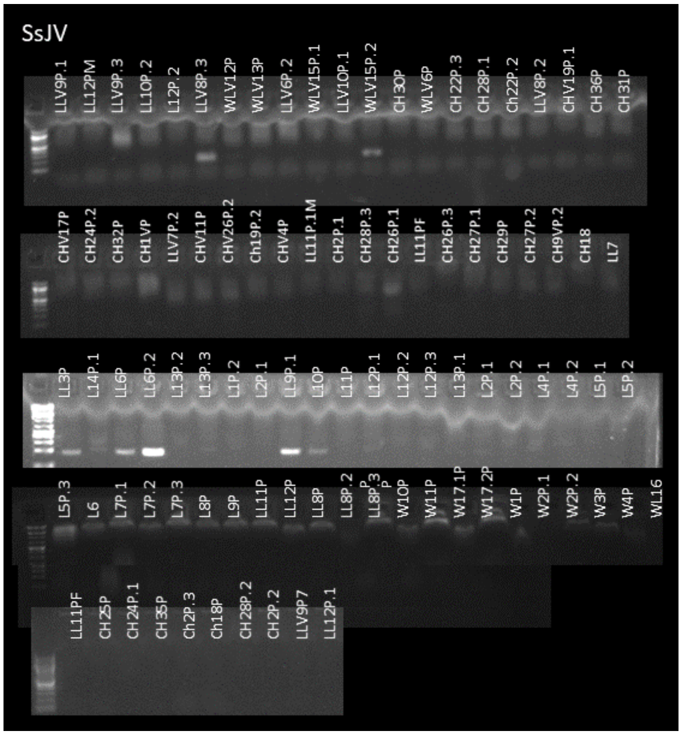

Figure S6: Detection of SsJV1 in plerocercoids from Cheney Lake (CL, 31 individuals), Loberg Lake (LL, 46 individuals) and Wolf lake (WL, 20 individuals). .1 .2 and .3 is used to label plerocercoids collected from the same stickleback host.

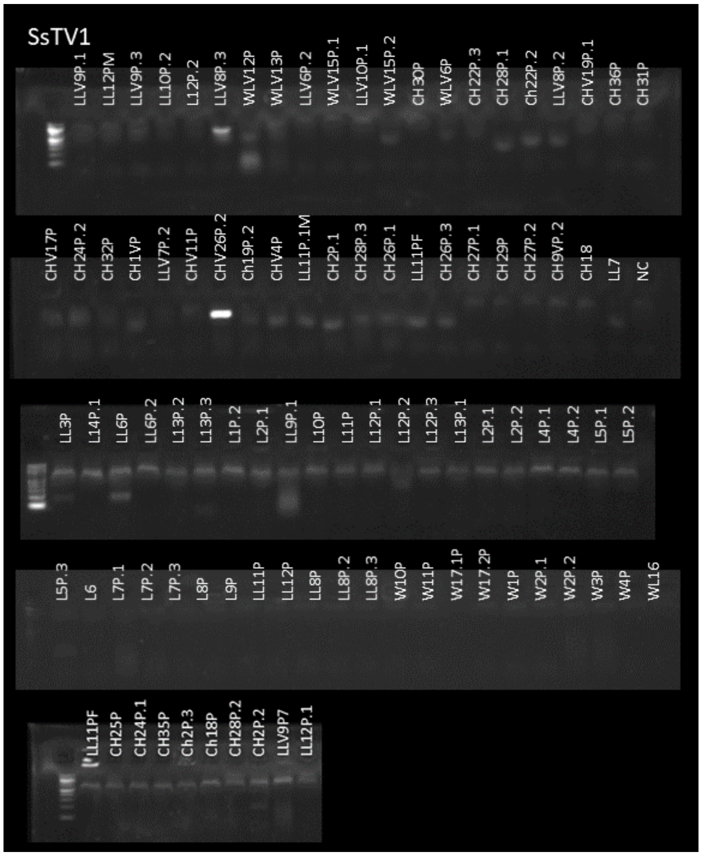

Figure S7: Detection of SsTV1 in plerocercoids from Cheney Lake (CL, 31 individuals), Loberg Lake (LL, 46 individuals) and Wolf lake (WL, 20 individuals). .1 .2 and .3 is used to label plerocercoids collected from the same stickleback host.

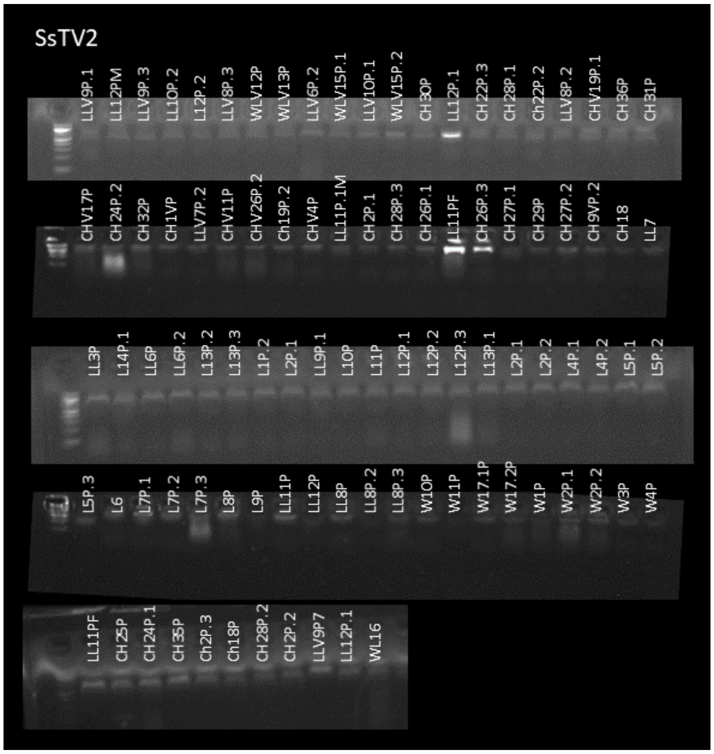

Figure S8: Detection of SsTV2 in plerocercoids from Cheney Lake (CL, 31 individuals), Loberg Lake (LL, 46 individuals) and Wolf lake (WL, 20 individuals). .1 .2 and .3 is used to label plerocercoids collected from the same stickleback host.

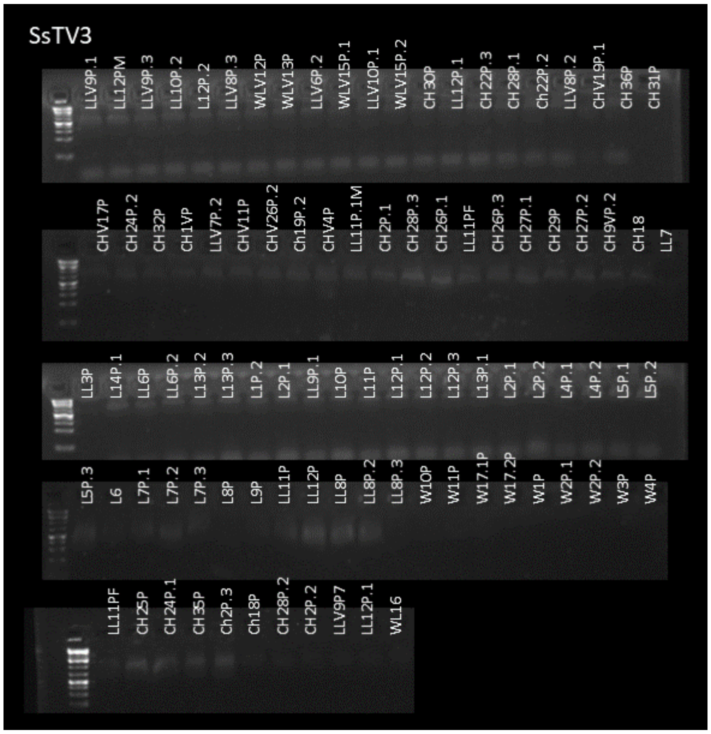

Figure S9: Detection of SsTV3 in plerocercoids from Cheney Lake (CL, 31 individuals), Loberg Lake (LL, 46 individuals) and Wolf lake (WL, 20 individuals). .1 .2 and .3 is used to label plerocercoids collected from the same stickleback host.

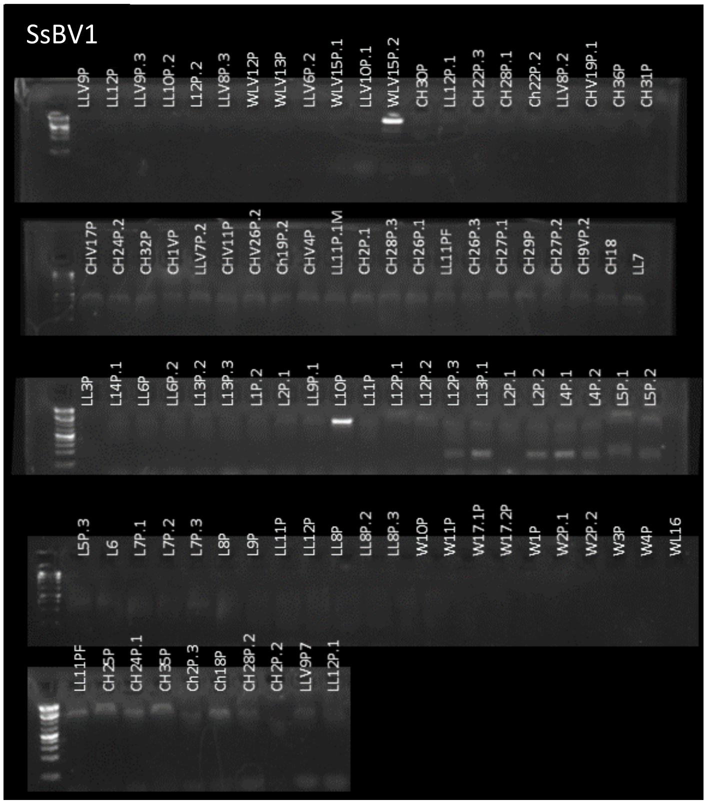

Figure S10: Detection of SsBV1 in plerocercoids from Cheney Lake (CL, 31 individuals), Loberg Lake (LL, 46 individuals) and Wolf lake (WL, 20 individuals). .1 .2 and .3 is used to label plerocercoids collected from the same stickleback host.

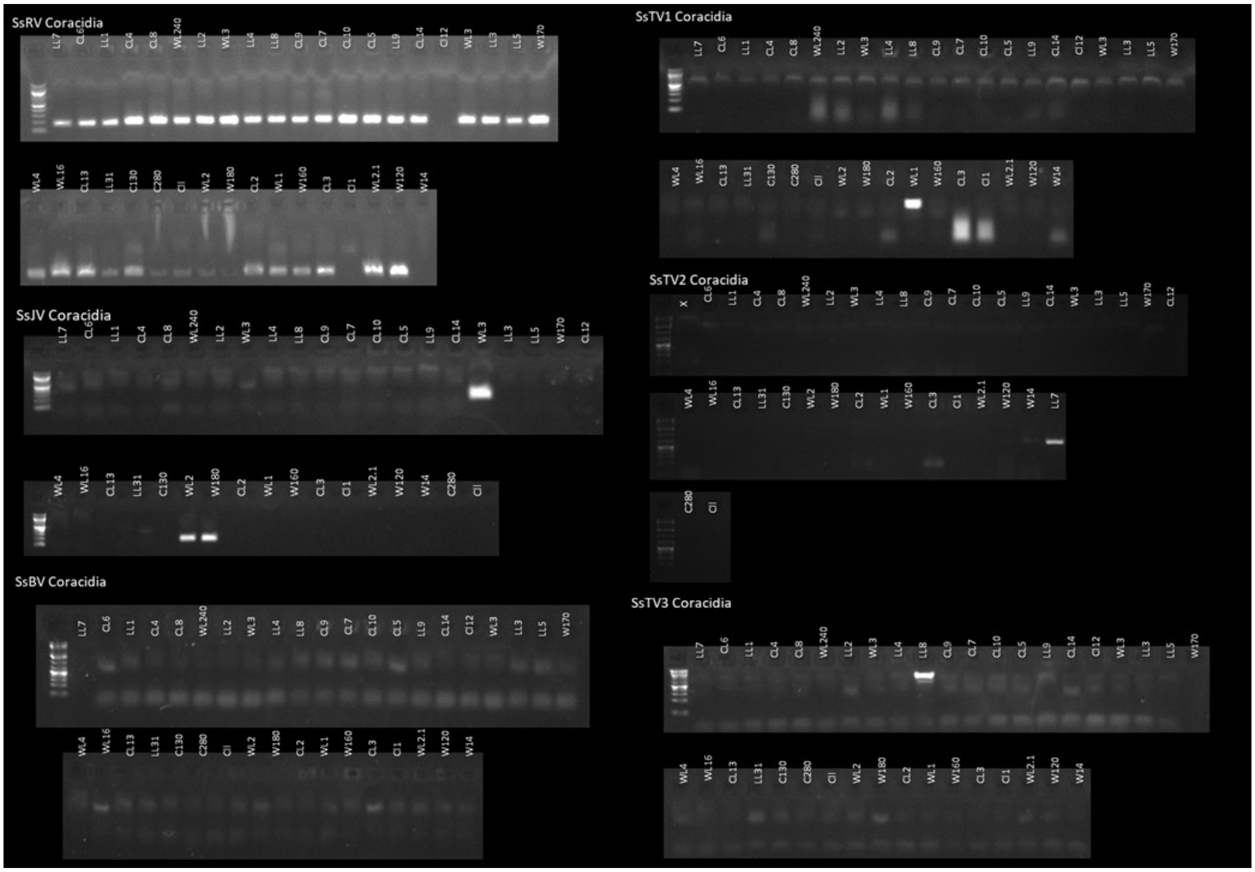

Figure S11: Detection of SsRV1, SsJV1, SsBV1, SsTV1, SsTV2 and SsTV3 in coracidia from 38 families produced *in vitro* from plerocercoids collected in Cheney Lake (CL, 16 families), Loberg Lake (LL, 9 families) and Wolf lake (WL, 13 families).

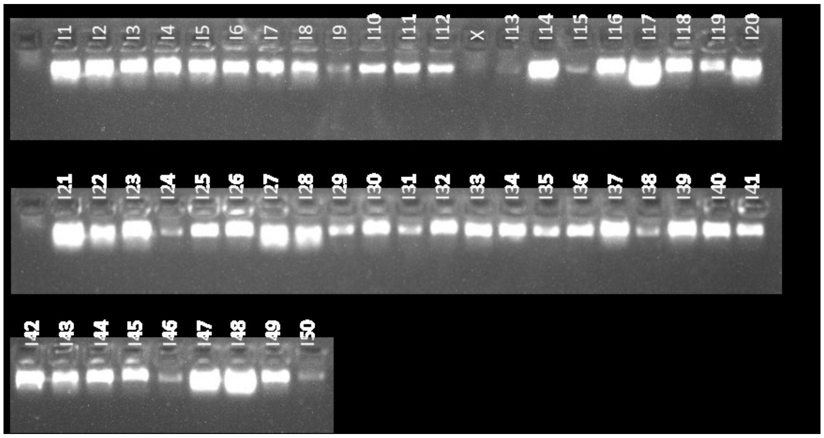

Figures S12: Detection of SsRV1 in procercoids following experimental infection of copepods with coracidia from the SsRV1(+) family WL1.

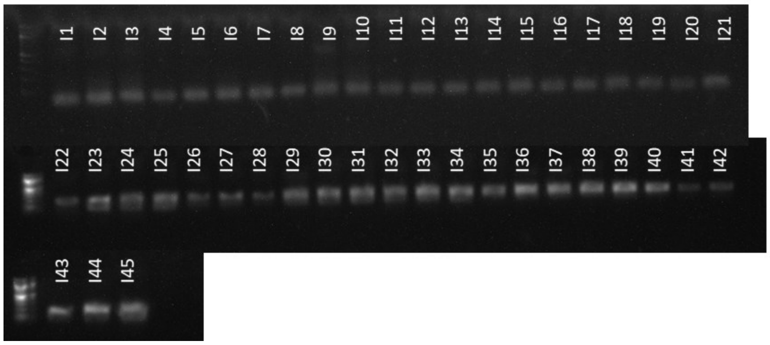

Figures S13: Detection of SsJV1 in procercoids following experimental infection of copepods with coracidia from the SsJV1(+) family WL240.

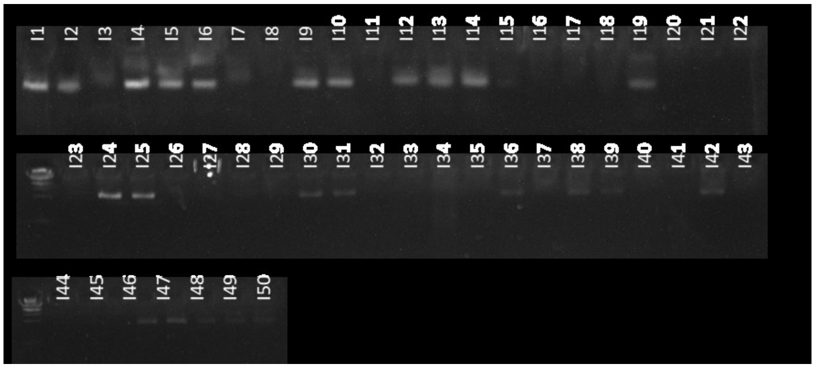

Figure S14: Detection of SsTV1 in procercoids following experimental infection of copepods with coracidia from the SsTV1(+) family WL1.

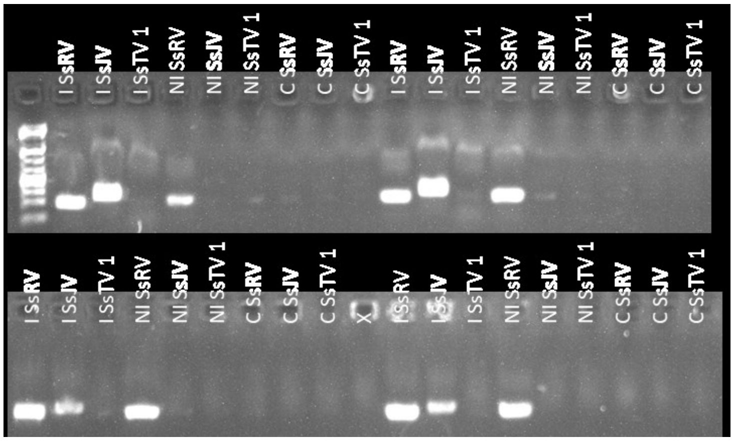

Figure S15: Detection of SsRV1, SsJV1, and SsTV1 in copepods experimentally infected by *S. solidus*. C: control non-exposed NI: exposed but non-infected I: infected.

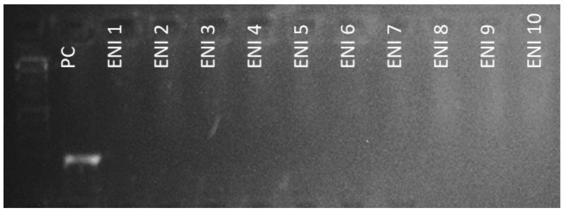

Figure S16: Detection of *S. solidus* in copepods exposed to *S. solidus* but not infected (ENI) and a positive control (PC).

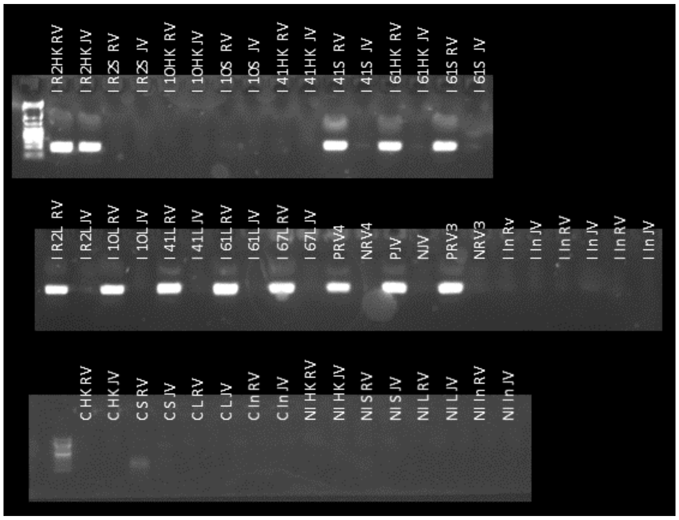

Figure S17: Detection of SsRV1 and SsJV1 in head kidney (HK), spleen (S), Liver (L), and intestine (In) tissues of Threespine sticklebacks experimentally infected by *S. solidus*. C: control non-exposed NI: exposed but non-infected I: infected. RV: SsRV1; JV: SsJV1

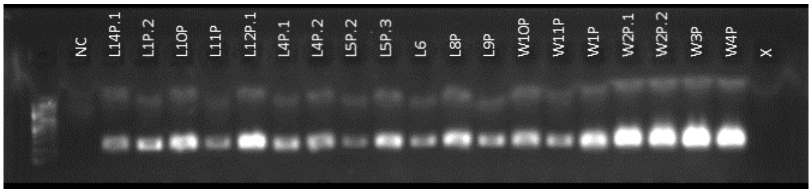

Figure S18: Detection of SsRV1 in liver tissue from field collected sticklebacks. The presence of SsRV1 in plerocercoids from corresponding fish individuals is provided in Figure 1. NC represents the negative control.

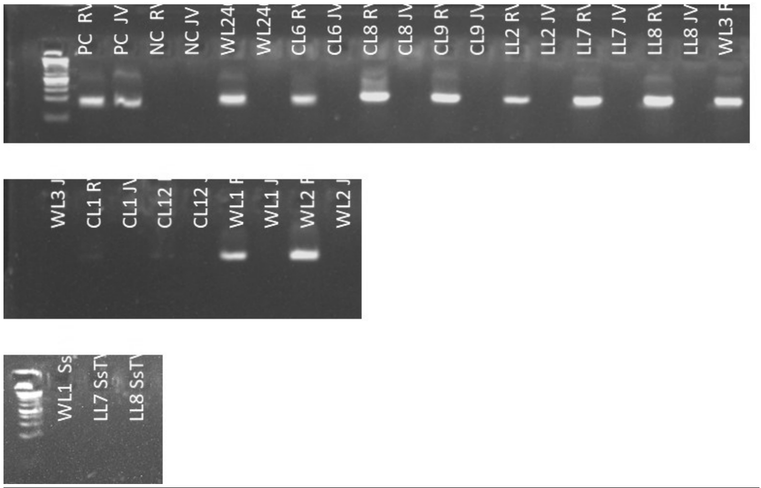

Figure S19: Detection of SsRV1, SsJV1, and SsTV1 in the culture medium used for in vitro breeding of *S. solidus* families. Presence of the corresponding viruses in coracidia of corresponding families is available in supplementary figure 2. Positive control (PC) and negative control (NC) were also included.

References

1. Ng TFF, Manire C, Borrowman K, Langer T, Ehrhart L, Breitbart M. Discovery of a novel single-stranded DNA virus from a sea turtle fibropapilloma by using viral metagenomics. Journal of Virology. 2009;83(6):2500-9.
2. Ng TFF, Suedmeyer WK, Wheeler E, Gulland F, Breitbart M. Novel anellovirus discovered from a mortality event of captive California sea lions. Journal of General Virology. 2009;90(5):1256-61.
3. Langmead B, Salzberg SL. Fast gapped-read alignment with Bowtie 2. Nature methods. 2012;9(4):357.
4. Andrews S. FastQC: a quality control tool for high throughput sequence data. Babraham Bioinformatics, Babraham Institute, Cambridge, United Kingdom; 2010.
5. Smyth J. Studies on tapeworm physiology: I. The cultivation of *Schistocephalus solidus* in vitro. Journal of Experimental Biology. 1946;23(1):47-70.
6. Wedekind C. The infectivity, growth, and virulence of the cestode *Schistocephalus solidus* in its first intermediate host, the copepod *Macrocyclops albidus*. Parasitology. 1997;115(3):317-24.
7. Lüscher A, Milinski M. Simultaneous hermaphrodites reproducing in pairs self-fertilize some of their eggs: an experimental test of predictions of mixed-mating and Hermaphrodite's Dilemma theory. Journal of Evolutionary Biology. 2003;16(5):1030-7.
8. Bolger AM, Lohse M, Usadel B. Trimmomatic: a flexible trimmer for Illumina sequence data. Bioinformatics. 2014;30(15):2114-20.
9. Roux S, Trubl G, Goudeau D, Nath N, Couradeau E, Ahlgren NA, et al. Optimizing de novo genome assembly from PCR-amplified metagenomes. PeerJ. 2019;7:e6902.

10. Bankevich A, Nurk S, Antipov D, Gurevich AA, Dvorkin M, Kulikov AS, et al. SPAdes: a new genome assembly algorithm and its applications to single-cell sequencing. Journal of computational biology. 2012;19(5):455-77.
11. Scotto–Lavino E, Du G, Frohman MA. 5′ end cDNA amplification using classic RACE. Nature protocols. 2006;1(6):2555.
12. Ahmadi E, Soleimanjahi H, Sadeghizadeh M, Teimoori A. Development of Poly (A)-Tailed Universal Reverse Transcription PCR Method for Sequence-Independent Amplification of Rearranged Rotavirus. Archives of Iranian Medicine (AIM). 2016;19(9).
13. Katoh K, Standley DM. MAFFT multiple sequence alignment software version 7: improvements in performance and usability. Molecular biology and evolution. 2013;30(4):772-80.
14. Walker PJ, Firth C, Widen SG, Blasdell KR, Guzman H, Wood TG, et al. Evolution of genome size and complexity in the Rhabdoviridae. PLoS pathogens. 2015;11(2).
15. Li C-X, Shi M, Tian J-H, Lin X-D, Kang Y-J, Chen L-J, et al. Unprecedented genomic diversity of RNA viruses in arthropods reveals the ancestry of negative-sense RNA viruses. 2015;4:e05378.
16. Shi M, Lin X-D, Chen X, Tian J-H, Chen L-J, Li K, et al. The evolutionary history of vertebrate RNA viruses. 2018;556(7700):197-202.
17. Shi M, Lin X-D, Tian J-H, Chen L-J, Chen X, Li C-X, et al. Redefining the invertebrate RNA virosphere. 2016;540(7634):539-43.
18. Capella-Gutiérrez S, Silla-Martínez JM, Gabaldón T. trimAl: a tool for automated alignment trimming in large-scale phylogenetic analyses. Bioinformatics. 2009;25(15):1972-3.
19. Lefort V, Longueville J-E, Gascuel O. SMS: smart model selection in PhyML. Molecular biology and evolution. 2017;34(9):2422-4.
20. Van der Veen I, Kurtz J. To avoid or eliminate: cestode infections in copepods. Parasitology. 2002;124(4):465-74.
21. Berger CS, Aubin-Horth N. An eDNA-qPCR assay to detect the presence of the parasite Schistocephalus solidus inside its threespine stickleback host. Journal of Experimental Biology. 2018;221(9):jeb178137.
22. Hébert FO, Grambauer S, Barber I, Landry CR, Aubin-Horth N. Transcriptome sequences spanning key developmental states as a resource for the study of the cestode *Schistocephalus solidus*, a threespine stickleback parasite. GigaScience. 2016;5(1):24.
23. Li H, Handsaker B, Wysoker A, Fennell T, Ruan J, Homer N, et al. Genome Project Data Processing Subgroup. 2009. The Sequence alignment/map (SAM) format and SAMtools. Bioinformatics.1000(25):16.
24. Quinlan AR, Hall IM. BEDTools: a flexible suite of utilities for comparing genomic features. Bioinformatics. 2010;26(6):841-2.
25. Li H. Aligning sequence reads, clone sequences and assembly contigs with BWA-MEM. arXiv preprint arXiv:13033997. 2013.
